# A Nanomodulator Enhances Radiotherapy‐Induced In Situ Cancer Vaccine by Promoting Antigen‐Presenting of Tumor‐Associated Macrophage

**DOI:** 10.1002/advs.202502876

**Published:** 2025-08-05

**Authors:** Xiu Zhao, Mengli Li, Jun Li, Yueying Han, Yu Gong, Zhenzhong Zhang, Jinjin Shi, Cheng‐Yun Jin, Junjie Liu, Pilei Si

**Affiliations:** ^1^ School of Pharmaceutical Sciences Zhengzhou University Zhengzhou 450001 China; ^2^ Henan Key Laboratory of Nanomedicine for Targeted Diagnosis and Therapy Zhengzhou University Zhengzhou 450001 China; ^3^ Key Laboratory of Advanced Drug Preparation Technologies Ministry of Education Zhengzhou 450001 China; ^4^ Department of Breast Surgery Henan Provincial People's Hospital Zhengzhou 450003 China

**Keywords:** in situ vaccine effect, nanomedicine, radiotherapy, tumor antigen presentation

## Abstract

Radiotherapy (RT) can induce an in situ vaccine effect by promoting the generation of tumor neoantigens, yet this effect is insufficient to elicit robust antitumor immune responses. Although the abundant tumor‐associated macrophages (TAMs) in tumor tissue, as members of antigen‐presenting cells, have been shown to capture antigens efficiently, the proteomic analysis, that TAMs is demonstrated exhibit up‐regulated cysteine protease in lysosomes that leads to tumor antigen degradation. Inhibiting cysteine protease activity can promote the antigen‐presenting of TAMs. Based on this, a nanomodulator (Ft‐E64/Hf@Lipo) is developed, combining radiosensitizer hafnium (Hf) and the cysteine protease inhibitor E64, which cooperatively reinvigorated the antigen presentation of TAMs. Ft‐E64/Hf@Lipo sensitized RT generated abundant tumor neoantigens, and then E64/antigens, along with the apoptotic tumor cells, trafficked to TAMs via efferocytosis. The reprogrammed TAMs with attenuated lysosomal function effectively presented tumor antigens and activated CD8^+^ T cells. In vivo studies demonstrated that the nanomodulator significantly enhanced systemic antitumor immune responses following RT, realizing excellent therapeutic efficacy against large, treatment‐resistant CT26 tumors in combination with anti‐PD‐1 therapy. The work provides a promising approach for enhancing the in situ vaccine effect of RT to improve its clinical benefits.

## Graphical abstract

A nanomodulator (Ft‐E64/Hf@Lipo) is developed, combining radiosensitizer hafnium (Hf) and the cysteine protease inhibitor E64, which cooperatively reinvigorated the antigen presentation of TAMs. Ft‐E64/Hf@Lipo sensitized‐RT generated abundant tumor neoantigens, and then E64/antigens, along with the apoptotic tumor cells, trafficked to TAMs via efferocytosis. The reprogrammed TAMs with attenuated lysosomal function effectively activated CD8^+^ T cells to boost the in situ vaccine effect of cancer radiotherapy.

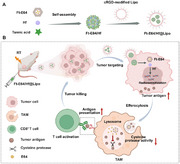

## 1 Introduction

Radiotherapy (RT) is the most common treatment for tumors in clinics, employed by ≈50%–60% of cancer patients.^[^ ^1^ ^]^ As a means of deeply penetrating and DNA damage‐generating stimulation, RT promotes tumor neoantigen generation, which alters the peptide repertoire available for effector T cell recognition and is conducive to stimulating a tumor‐specific systemic immune response. Studies have demonstrated that RT can activate an in situ vaccine response,^[^ ^2^ ^]^ and multiple clinical trials have demonstrated that RT augments response rates to immune checkpoint blockade therapy.^[^ ^3^ ^]^ However, the inefficient antigen presentation greatly weakens the in situ vaccine effect of RT.^[^ ^4^ ^]^ Consequently, the abscopal response following RT is less than 1% in clinical practice.^[^ ^5^ ^]^

Although the antigen‐presenting process is traditionally ascribed to dendritic cells (DCs), the population of DCs in TME is typically low.^[^ ^6^ ^]^ The tumor‐associated macrophages (TAMs), as members of antigen‐presenting cells, are the most abundant immune cells in the tumor microenvironment (TME). Mounting evidence has shown that TAMs are more phagocytic than DCs,^[^ ^7^ ^],^ and their mediated invalid antigen processing is a key factor that leads to tumor immune escape.^[^ ^8^ ^]^ Currently, various treatment strategies, including inducing TAMs' apoptosis or repolarizing them into immunostimulatory M1 phenotype, have been developed in preclinical research and clinical trials.^[^ ^9^ ^]^ However, the inherent disadvantage of consuming TAM is the loss of its natural immune‐stimulating effect as the main professional antigen‐presenting cell. In addition, affected by the immunosuppressive TME, the reprogrammed M1 TAMs may be repolarized into an M2‐like phenotype. Thus, inhibiting the underlying principles responsible for antigen degradation by TAMs and harnessing them to directly activate CD8^+^ T cells is an efficient and attractive strategy for enhanced radio‐immunotherapy.

**Scheme 1:**
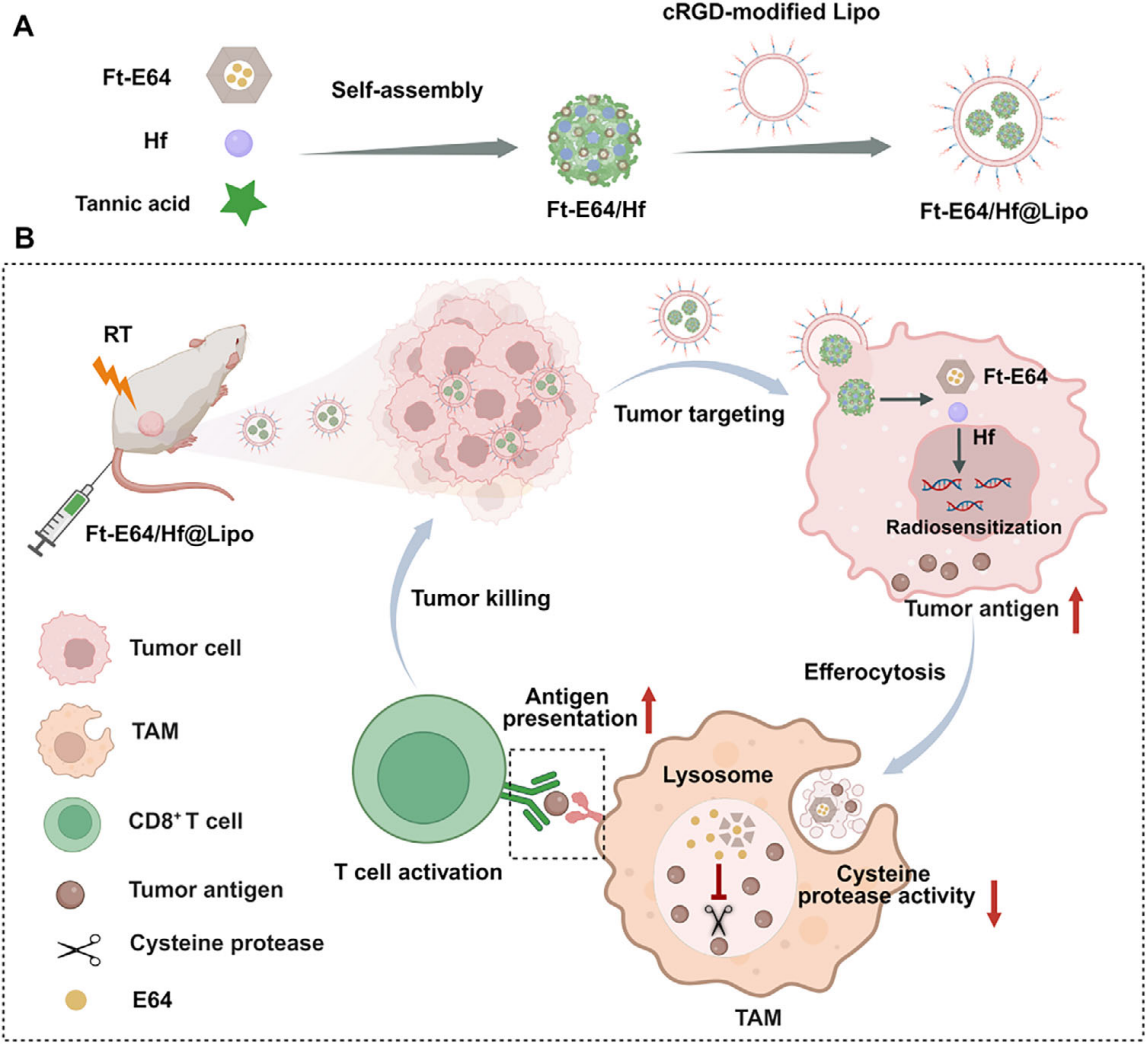
Schematic of Ft‐E64/Hf@Lipo enhanced radio‐immunotherapy. A) Schematic illustration of the synthetic procedure for Ft‐E64/Hf@Lipo. B) Schematic illustration of Ft‐E64/Hf@Lipo to boost the in situ vaccine effect of cancer radiotherapy. Ft‐E64/Hf@Lipo can selectively target tumor cells, radiosensitizer Hf augments DNA damage to generate abundant tumor antigens; and then Ft‐E64, along with the tumor antigens, is phagocytosed by tumor‐associated macrophage (TAMs) through efferocytosis. The released E64 inhibits the lysosomal function of TAMs, enabling them to effectively present tumor antigens and activate antigen‐specific CD8^+^ T cells for enhanced radio‐immunotherapy.

Here, through preliminary exploration, we found that TAMs highly expressed lysosomal cysteine protease, which lead to tumor antigen degradation. The small‐molecule cysteine protease inhibitor E64 could reduce antigen degradation, restore the antigen presentation of M2 TAMs, and improve the in vivo antitumor efficiency of RT. In addition, considering that the insufficient X‐ray deposition within tumor tissues limits the effective production of tumor antigens, hafnium (Hf)‐based radiosensitizers have entered clinical trials.^[^ ^10^ ^]^ Here, we developed an integrated radiation nanomodulator (Ft‐E64/Hf@Lipo) for increasing tumor neoantigen generation and meanwhile promoting the antigen presentation of TAMs to enhance cancer radio‐immunotherapy. Ft‐E64/Hf@Lipo is constructed by the assembly of E64‐loaded ferritin (Ft) and Hf, with further modification of CRGD‐modified fusogenic liposomes. Ft‐E64/Hf could effectively enter the cytoplasm of tumor cells through the membrane fusion pathway and then dissociate under intracellular glutathione (GSH). Under X‐ray irradiation, Hf‐mediated radiosensitization promoted the generation of abundant tumor‐specific antigens, which, along with the Ft‐E64, are phagocytosed by TAMs through efferocytosis. E64 reprogrammed the lysosome of TAMs to endow it with a suitable antigen peptide‐generating environment, thus effectively presenting tumor antigens to activate CD8^+^ T cells‐dependent antitumor immunity (**Scheme** **1**B). The constructed radiation nanomodulator effectively facilitated the regression of primary and distal tumors, and remained effective in large colorectal cancer.

## 2 Results

### 2.1 TAMs Exhibit High Lysosomal Cysteine Proteases to Degrade Antigens

**Figure 1:**
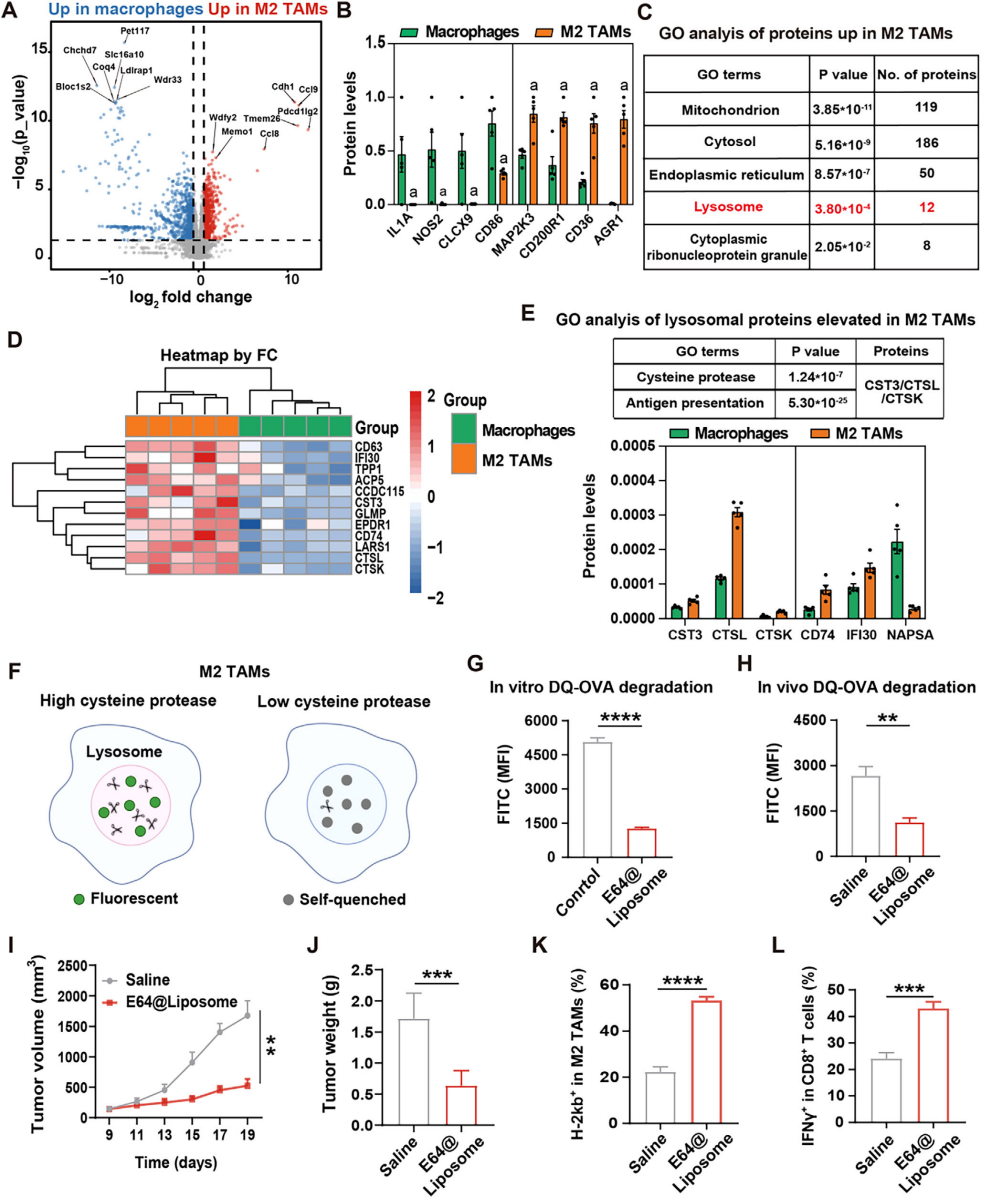
Lysosomal cysteine proteases play a specific role in antigen cross‐presentation by TAMs. A) 4D‐DIA proteomics analysis of whole‐cell lysates from IL‐4/IL‐13 treated BMDMs (simulating M2 TAMs) and LPS/IFN‐γ‐treated BMDMs (simulating macrophages with antigen‐presenting function) (*n* = 5). B) Associated protein levels in macrophages and M2 TAMs (*n *= 5). C) Top pathways from gene ontology (GO) analysis of proteins elevated in M2 TAMs, obtained from five samples. D) Heatmap of lysosomal protein levels in M2 TAMs and macrophages (*n *= 5). E) The top two pathways identified in GO analysis for upregulated lysosomal proteins in M2 TAMs are presented above. The levels of cysteine proteases in macrophages/M2 TAMs were quantified, as shown below (*n* = 5). F) Assay scheme of DQ‐OVA degradation assay. The results of G) in vitro and H) in vivo DQ‐OVA antigen degradation assay (*n* = 3). I) Tumor growth curves after different treatments (*n *= 6). J) The sacrificed tumor weights on the 19th day (*n* = 6). The quantification analysis of (K) MHC‐I expression on M2 TAMs and (L) CD8^+^ T cell activation (*n* = 3). Data are means ± SD. **p < 0.01, ***p < 0.001 and ****p < 0.0001 determined by Student's t‐test.

To elucidate the mechanism of invalid antigen processing by TAMs, we conducted a comparative analysis with macrophages possessing antigen‐presenting capability. Bone marrow stem cells were extracted from mice and cultured to differentiate into bone marrow‐derived macrophages (BMDMs). BMDMs pretreated with IL‐4 and IL‐13 were applied to simulate TAMs with M2‐phenotype, and LPS/IFN‐γ‐activated BMDMs simulated macrophages with antigen‐presenting function. The 4D‐Data independent acquisition (4D‐DIA) proteomics assessment of cell lysates derived from M2‐activated BMDMs revealed 827 proteins were significantly upregulated (with a false discovery rate of <5%) (**Figure** **1**A). Notably, many of these proteins were previously characterized to be associated with the M2‐phenotype, including ARG1 and YM1 (Figure 1B). Bioinformatics analysis indicated that the upregulated proteins of M2 TAMs were significantly enriched in the cytoplasm (Figure S1, Supporting Information). Further analysis of the cytoplasm showed significant enrichment in both the mitochondrion and cytosol, highlighting their essential roles in cellular energy production and metabolic regulation (Figure 1C; Figure S2, Supporting Information). Notably, lysosomal proteins were also found to be up‐regulated in M2 TAMs (Figure 1C,D). The lysosome is the primary organelle responsible for processing and presenting antigens,^[^ ^11^ ^]^ thus, we made further bioinformatics analysis on the lysosome. An enrichment of antigen presentation and cysteine proteases, such as CTSL, CST3, and CTSK, was observed in M2 TAMs (Figure 1E). It's reported that cysteine proteases are capable of completely digesting antigenic peptides.^[^ ^12^ ^]^ Therefore, we speculated that the enriched lysosomal cysteine proteases in TAMs played a vital role in hindering antigen presentation. Subsequently, using a DQ Ovalbumin (DQ‐OVA) degradation assay, we tested whether the small‐molecule cysteine protease inhibitor, E64, could restore the antigen presentation of M2 TAMs. DQ‐OVA is a self‐quenching OVA conjugate that exhibits fluorescence during protein degradation (Figure 1F). A significant reduction of OVA degradation was observed in M2 TAMs pre‐treated with E64@liposome (Figure 1G).

Next, we established a CT26 tumor‐bearing mouse model to investigate the in vivo anti‐tumor activity of E64@liposome. As depicted in Figure 1I,J, and Figure S3, Supporting Information, the tumor inhibition rate was improved by 61.2% after E64@liposome treatment, and the body weight of mice didn't change significantly. TAMs collected from tumor tissue were incubated with DQ‐OVA (10 µg mL^−1^). We found that the antigen degradation ability of TAMs was reduced 2.4‐fold (Figure 1H). Moreover, the flow cytometry analysis results revealed that, after E64@liposome treatment, there was a 27.5% increase in MHC‐I molecule expression on M2 TAMs (Figure 1K), and the population of tumor‐infiltrating IFN‐γ^+^ CD8^+^ T cells was improved by 18.5% (Figure 1L). Collectively, these results suggested that M2 TAMs exhibited high lysosomal cysteine proteases to degrade antigens, and suppressing the cysteine protease activity could promote the antigen cross‐presentation and thus attenuate tumor growth.

### 2.2 Preparation and Characterization of Ft‐E64/Hf@Lipo Nanomodulator

**Figure 2:**
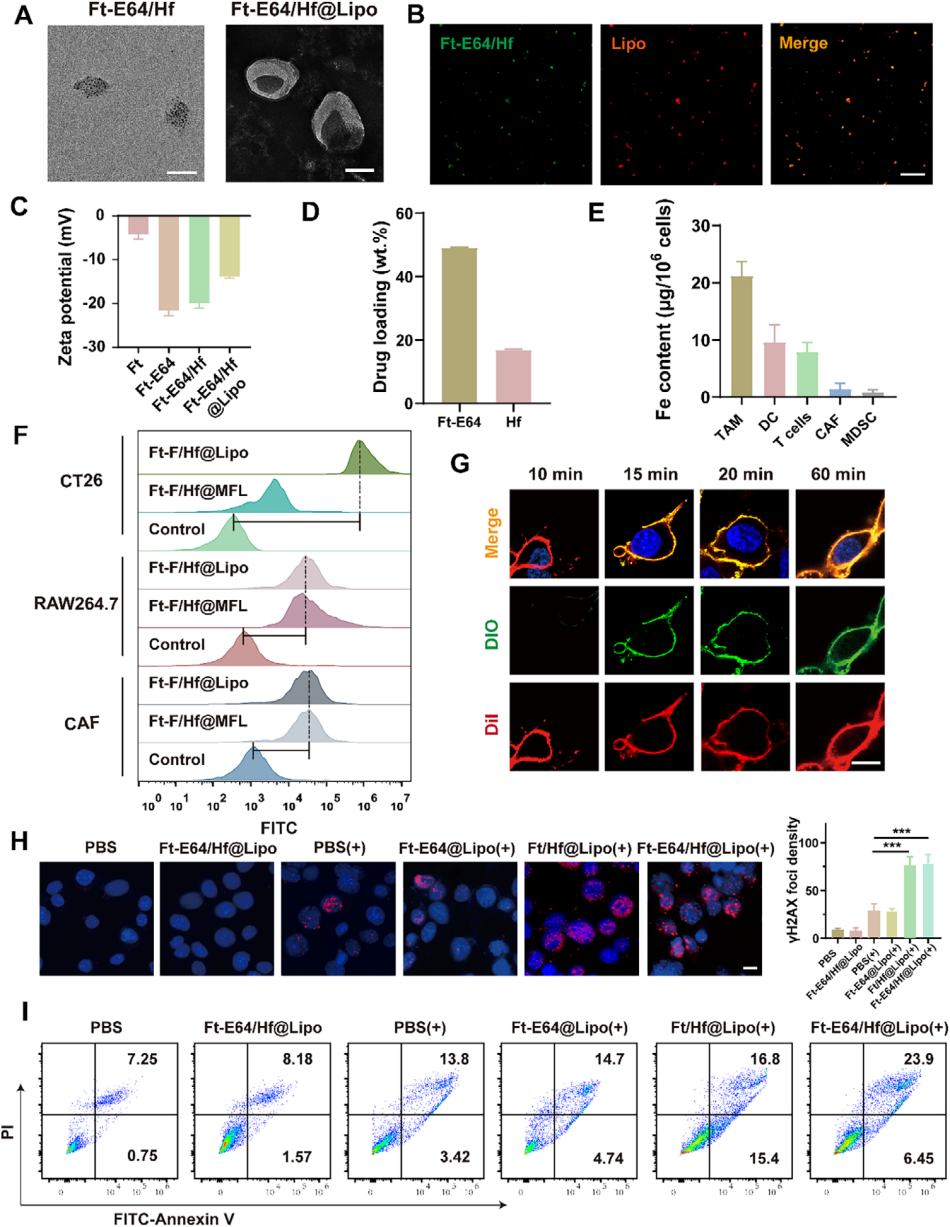
Preparation of Ft‐E64/Hf@Lipo that enhances RT‐induced DNA damage and tumor apoptosis. A) TEM images of Ft‐E64/Hf and Ft‐E64/Hf@Lipo. Scale bars: 100 nm. B) CLSM images of Ft‐E64/Hf@Lipo (DiI‐labeled Lipo, red; FITC‐labeled Ft‐E64/Hf, green). Scale bar: 5 µm. C) Zeta potentials of various preparations (*n* = 3). D) Loading amounts of Ft‐E64 and Hf in Ft‐E64/Hf (*n* = 3). E) ICP‐MS analysis of Fe content across different cell populations within the TME after Ft‐E64/Hf@Lipo‐combined RT (*n* = 3). F) Flow cytometry analysis of the uptake capacity of Ft‐F/Hf@Lipo by different cells. G) Study on the fusion process between Lipo (labeled with DIO) and CT26 cell membrane (labeled with DiI). Scale bar: 10 µm. H) Representative immunofluorescent images (Scale bar: 10 µm) and the quantitative analysis of γ‐H2AX in CT26 cells after different treatments (*n* = 3). I) Flow cytometer analysis of the CT26 cells' apoptotic ratios. “+” represented RT. Data are means ± SD. ***p < 0.001 determined by Student's t‐test.

To enhance the in situ vaccine effect of RT, an integrated radiation nanomodulator (Ft‐E64/Hf@Lipo) was constructed as illustrated in Scheme 1A. Initially, E64 was loaded into Ft with high biosafety (denoted as Ft‐E64), which exhibited an average particle of ≈10 nm, similar to that of Ft (Figure S4, Supporting Information). The significantly reduced Zeta potential demonstrated the successful loading of E64 (Figure 2C). To further enhance tumor neoantigen generation, high‐Z metal Hf was selected as a radiosensitizer. Ft‐E64, Hf, and tannic acid (TA) were self‐assembled based on the multiple interactions of polyphenols and proteins, and the metal‐phenolic coordination. The constructed self‐assembled nanoparticles are denoted as Ft‐E64/Hf. As displayed in the transmission electron microscopy (TEM) image (**Figure** **2**A) and dynamic light scattering (DLS) analysis (Figure S5, Supporting Information), Ft‐E64/Hf nanoassembly exhibited an average particle size of ≈90 nm, and the uniform distribution of Hf elements further confirmed its successful preparation (Figure S6, Supporting Information). Quantitative results demonstrated that the loading amount of Ft‐E64 and Hf was 49.2 wt.% and 16.7 wt.%, respectively (Figure 2D). Subsequently, Ft‐E64/Hf nanoparticles were coated with cRGD peptide‐functionalized fusogenic liposomes (Lipo) for effectively delivering them to the cytoplasm of tumor cells. As displayed in Figure 2A, a distinct membrane coating was observed on the surface of Ft‐E64/Hf@Lipo with a particle size of ≈120 nm. Moreover, the confocal laser scanning microscopy (CLSM) image also displayed that Lipo exhibited co‐localization with Ft‐E64/Hf (Figure 2B). These results collectively indicated the successful preparation of Ft‐E64/Hf@Lipo. In addition, Ft‐E64/Hf@Lipo had excellent stability in a physiological environment (Figure S7, Supporting Information). It's reported that GSH in the cytosol can trigger TA‐protein nanoassembly depolymerization through competitive supramolecular interactions.^[^ ^13^ ^]^ As expected, the particle size of Ft‐E64/Hf exhibited a gradual decrease in conjunction with an elevated GSH concentration, and complete degradation of Ft‐E64/Hf occurred under 10 mM GSH (Figure S8A, Supporting Information). Moreover, a significant release of Hf from Ft‐E64/Hf was confirmed through inductively coupled plasma mass spectrometry (ICP‐MS) (Figure S8B, Supporting Information). Subsequently, we evaluated the E64 release in a simulated lysosomal acidic environment, with FITC serving as a substitute. The findings demonstrated that the drug release ratio reached 65.6% at pH 5.5 (Figure S8C, Supporting Information), which was conducive for E64 to inhibit lysosomal activity and promote the antigen‐presenting by TAMs.

### 2.3 Ft‐E64/Hf@Lipo Augments RT‐Induced DNA Damage and Tumor Apoptosis

Subsequently, we prepared FITC‐labeled Ft/Hf (Ft‐F/Hf), wherein FITC instead of E64, to investigate the targeting ability of Ft‐E64/Hf@Lipo toward different cells. Compared to macrophages and fibroblasts, the uptake of Ft‐F/Hf@Lipo by CT26 tumor cells was significantly enhanced, and Ft‐F/Hf@Lipo exhibited more efficient internalization by CT26 cells compared with that without cRGD modification (Figure 2F), suggesting that Ft‐E64/Hf@Lipo could effectively target CT26 tumor cells via the RGD peptide. Following a 15 min co‐incubation with CT26 cells, the membrane fusion between Lipo and CT26 cells was observed by CLSM (Figure 2G), and Ft‐E64/Hf was mainly localized in the cytosol (Figure S9A, Supporting Information). In addition, various endocytosis inhibitors had no significant effect on the uptake of Ft‐E64/Hf@Lipo (Figure S9B, Supporting Information). These results indicated that Ft‐E64/Hf@Lipo mainly entered CT26 cells through the membrane fusion pathway. Ft‐E64/Hf@Lipo showed no obvious cytotoxicity to CT26 cells without X‐ray irradiation (Figure S10, Supporting Information). Next, we investigated the radiosensitization effect of Ft‐E64/Hf@Lipo by detecting the DNA damage under irradiation. As shown in Figure 2H, compared to PBS (+) group, (+) represented 6 Gy X‐ray irradiation, the fluorescence intensity of γ‐H2AX in CT26 cells treated with Ft/Hf@Lipo (+) and Ft‐E64/Hf@Lipo (+) containing radiosensitizer Hf significantly increased by 2.6‐fold and 2.7‐fold, respectively, the effective DNA damage was beneficial for generating abundant tumor antigens. Moreover, when CT26 cells were treated with either Ft‐E64/Hf@Lipo or Ft/Hf@Lipo under 6 Gy irradiation, the apoptosis ratios were remarkably up to 32.2% and 30.4% (Figure 2I), respectively. The above results indicated that Hf‐mediated radiosensitization effectively augmented DNA damage and induced extensive tumor cell apoptosis. As expected, the high level of phosphatidylserine (PS) molecules, an “eat me” signal, was observed on the CT26 cells treated with Ft/Hf@Lipo (+) and Ft‐E64/Hf@Lipo (+) (Figure S11, Supporting Information), which can be recognized by phagocytic receptors present on macrophages, thereby facilitating efferocytosis.^[^ ^14^ ^]^ Next, we detected the distribution of Ft‐E64 across different cell populations within the TME through flow sorting and ICP‐MS analysis. As displayed in Figure 2E, the Fe content in TAMs significantly increased to 21.2 µg 10^−6^ cells, indicating a preferential accumulation of Ft‐E64 in TAMs after Ft‐E64/Hf@Lipo and RT treatment, which may be attributed to the efferocytosis of macrophages.

### 2.4 Ft‐E64/Hf@Lipo Enhanced RT Promotes the Antigen Presentation by M2 TAMs and Activates CD8^+^ T Cells In Vitro

**Figure 3:**
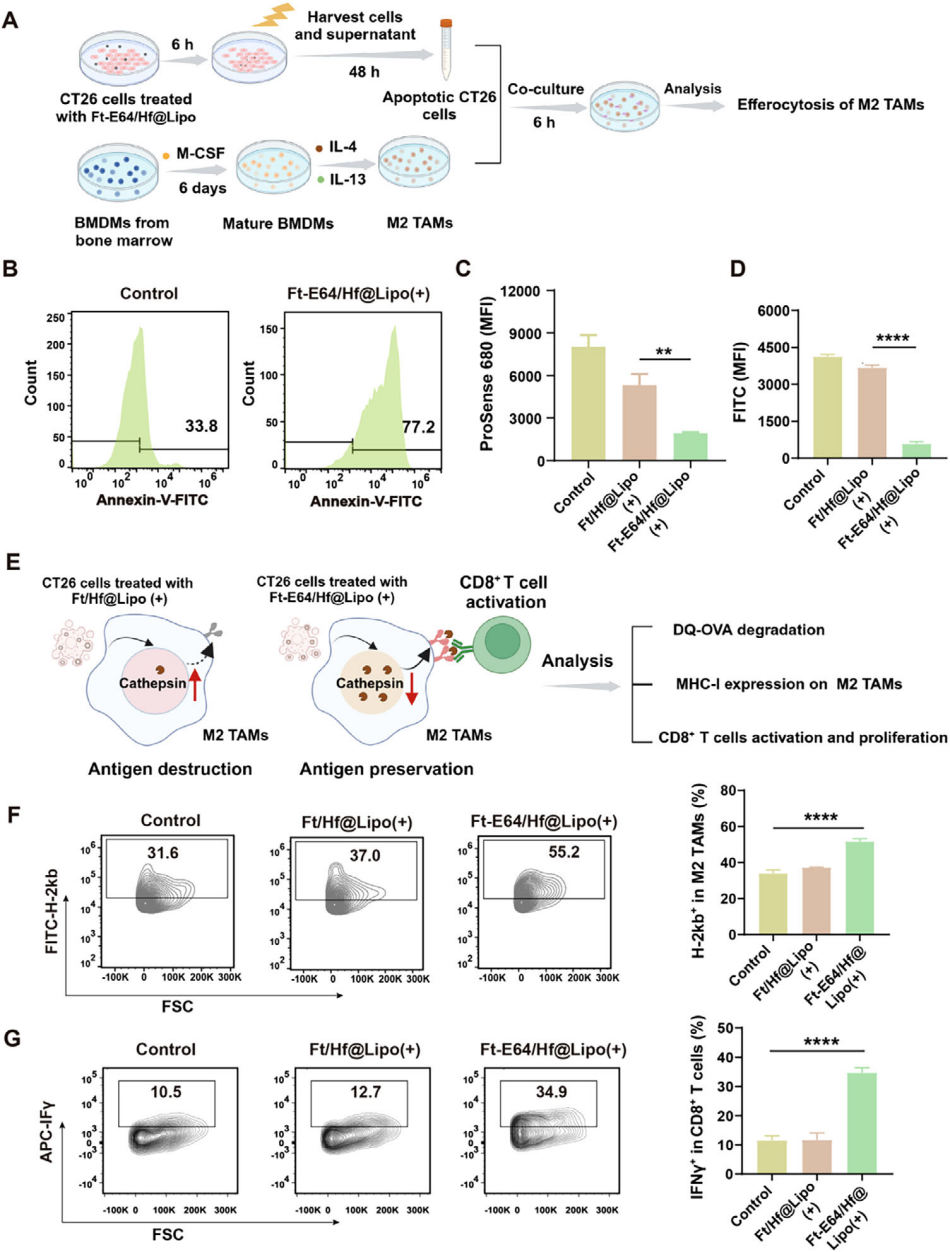
Ft‐E64/Hf@Lipo modulates the antigen presentation by M2 TAMs. A) The experimental design involves the efferocytosis of M2 TAMs to apoptotic CT26 cells. B) Flow cytometry analysis of M2 TAMs engulfing CT26 cells pre‐treated with Ft‐E64/Hf@Lipo (+). C) Cysteine protease activity of M2 TAMs after different treatments (*n* = 3). D) The result of the DQ‐OVA antigen degradation assay after different treatments (*n* = 3). E) Illustration of Ft‐E64/Hf@Lipo (+) treatment restoring the antigen presentation and CD8^+^ T cell activation capacity of M2 TAMs. Representative flow cytometry images and the quantification analysis of (F) MHC‐I expression on M2 TAMs, and (G) CD8^+^ T cell activation (*n* = 3). “+” represented RT. Data are means ± SD. **p < 0.01, ***p < 0.001, ****p < 0.0001 determined by Student's t‐test.

Encouraged by the above results, we next investigated the phagocytosis of TAMs on apoptotic CT26 cells in vitro. CT26 cells treated with Ft‐E64/Hf@Lipo and RT were co‐incubated with M2 TAMs and the specific experimental design was illustrated in **Figure** **3**A. We found that amounts of Annexin V‐FITC‐labeled apoptotic CT26 cells were engulfed by M2 TAMs (Figure 3B). Next, the ProSense 680 probe, a near‐infrared fluorescent indicator for proteolytic activation, was applied to detect the lysosomal cysteine protease activity of M2 TAMs. As presented in Figure 3C, when M2 TAMs engulfed apoptotic CT26 cells pretreated with Ft‐E64/Hf@Lipo (+), the lysosomal cysteine protease activity significantly decreased by 2.7‐fold compared with Ft/Hf@Lipo (+) treatment group, indicating that the E64 delivered to M2 TAMs via efferocytosis effectively reduced cysteine protease content within lysosomes of M2 TAMs. And a significant reduction of OVA degradation was observed (Figure 3D). Next, we further evaluated the antigen presentation and CD8^+^ T cell activation capacity of M2 TAMs as displayed in Figure 3E. Compared to the control group, the expression level of MHC‐I on M2 TAMs in the Ft‐E64/Hf@Lipo (+) group was significantly increased by 19.5% (Figure 3F). Subsequently, the activation of cytotoxic CD8^+^ T cells upon the immunomodulation effect of Ft‐E64/Hf@Lipo was evaluated in vitro. As presented in Figure 3G, compared to the control group (M2 TAMs alone), the Ft‐E64/Hf@Lipo (+) treatment group showed a significant increase of 23.0% in the number of IFN‐γ^+^CD8^+^ T cells. These findings suggested that Ft‐E64/Hf@Lipo‐mediated immunomodulation effectively promoted the antigen presentation of M2 TAMs, thus enhancing the activation of CD8^+^ T cells.

### 2.5 Ft‐E64/Hf@Lipo Enhanced RT Suppresses Primary and Distant Tumor Growth Through Adaptive Immune Activation

**Figure 4:**
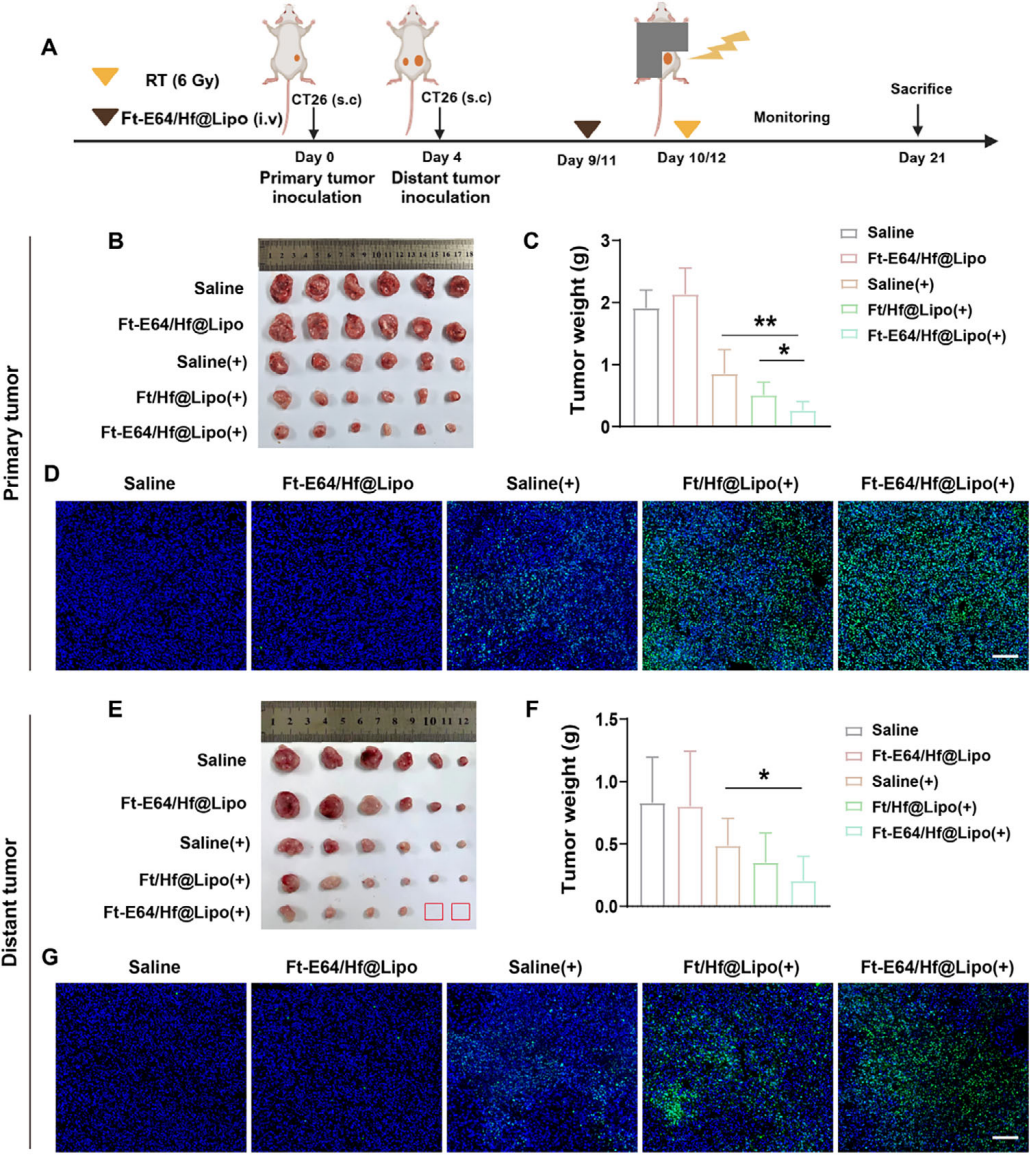
In vivo antitumor effect of Ft‐E64/Hf@Lipo‐enhanced RT on mice with a CT26 bilateral tumor model. A) Schematic illustration of the tumor inoculation and therapeutic schedule. Representative photos of the (B) primary and (E) distant tumor after various treatments (*n* = 6). Weights of the sacrificed (C) primary and (F) distant tumor on the 21st day (*n* = 6). TUNEL staining of the (D) primary and (G) distant tumor section after different treatments. Scale bars: 100 µm. “+” represented RT. Data are means ± SD. *p < 0.1, **p < 0.01 determined by Student's t‐test.

Next, a CT26 bilateral tumor‐bearing mouse model was established to assess the in vivo anti‐tumor effect of Ft‐E64/Hf@Lipo‐enhanced RT. As illustrated in **Figure** **4**A, the mice were intravenously administered Ft‐E64/Hf@Lipo twice at an interval of 48 h. At 12 h post‐injection, only the primary tumor in the RT groups was treated with 6 Gy irradiation, and the left distant tumors were not irradiated. As displayed in Figure 4B,C,E,F, in combination with Ft‐E64/Hf@Lipo, RT significantly inhibited the growth of primary and the un‐irradiated distant tumors; the tumor inhibition rate increased by 31% and 34% compared to the individual RT group, respectively. Moreover, Ft‐E64/Hf@Lipo (+) induced the most cell apoptosis in the primary and distant tumors (Figure 4D,G). These results collectively demonstrated that Ft‐E64/Hf@Lipo could effectively improve the anti‐tumor effect of RT. The body weight of mice didn't change significantly. H&E staining of main organs, blood routine, and blood biochemical tests further indicated that Ft‐E64/Hf@Lipo had no obvious damage to mice, indicating its good biosafety (Figure S12, Supporting Information). Moreover, following the inoculation of CT26 tumor cells on the control flank of mice bearing primary CT26 or B16 tumors, the secondary CT26 tumor growth of mice bearing primary CT26 tumor treated with Ft‐E64/Hf@Lipo (+) treatment was significantly delayed, while slight secondary CT26 tumor suppression was observed on the mice bearing primary B16 tumors (Figures S13A–C, Supporting Information), further indicating that Ft‐E64/Hf@Lipo (+) induced tumor suppression was antigen‐specific. In addition, the body weight of mice had no obvious change (Figures S13D, Supporting Information).

**Figure 5:**
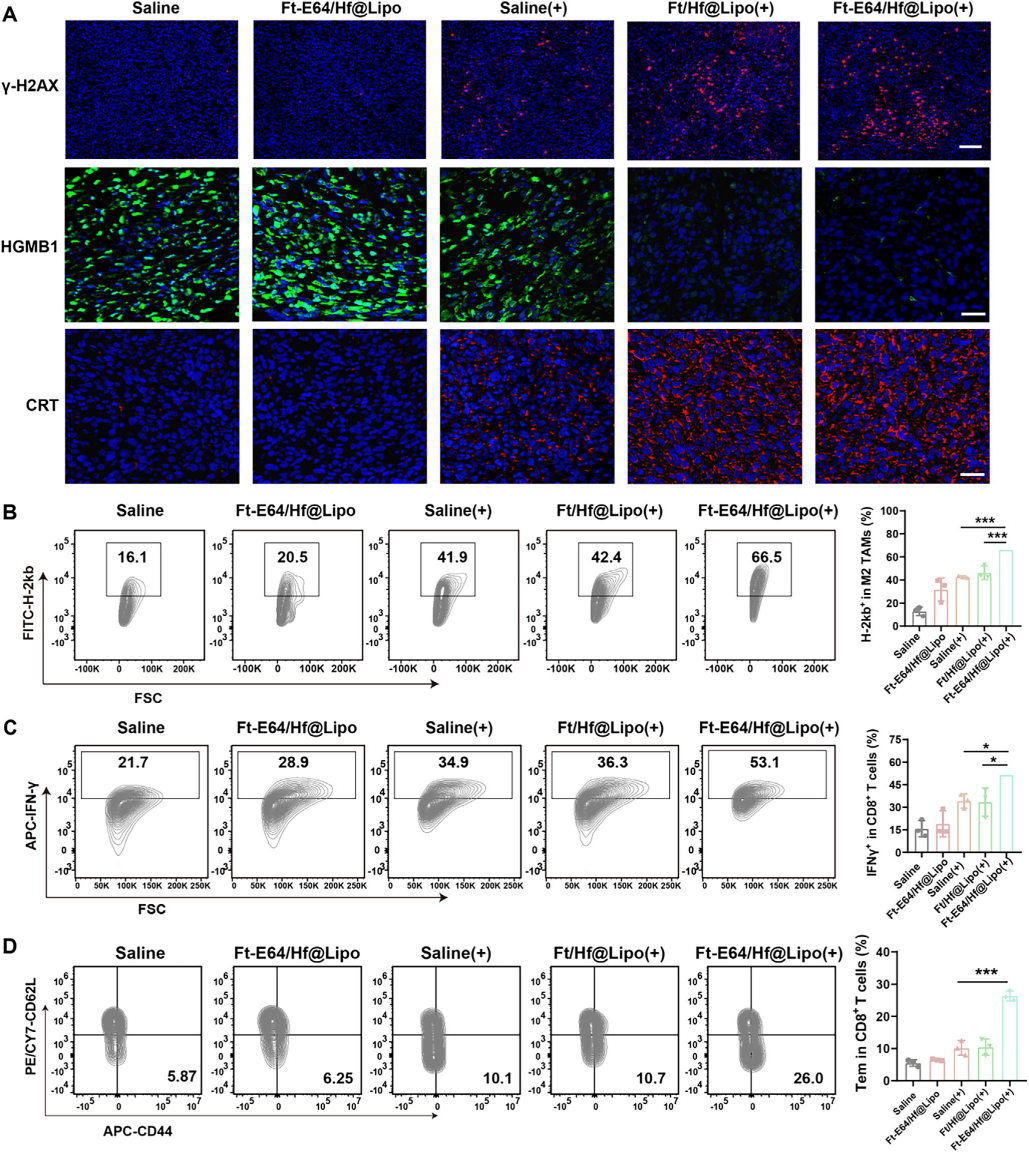
Systemic immune activation of Ft‐E64/Hf@Lipo enhanced radioimmunotherapy. A) The immunofluorescence analysis of γH2AX (Scale bar: 100 µm), CRT (Scale bar: 25 µm), and HMGB1 (Scale bar: 25 µm) in the primary tumor tissue extracted from mice. B) Representative flow cytometry images and the quantification analysis of MHC‐I molecule expression on M2 TAMs (*n* = 3). C) Flow cytometry analysis of the IFN‐γ^+^ CD8^+^ T cells in the primary tumor (*n* = 3). D) Effector memory CD8^+^ T cells in the spleen of mice after various treatments. Data are means ± SD. (*n* = 3). *p < 0.1, ***p < 0.001 determined by Student's t‐test.

To comprehend the potential anti‐tumor mechanism underlying Ft‐E64/Hf@Lipo combined RT, the primary tumor after the second round of treatment was collected. First, DNA damage was detected. Compared with RT alone, the tumors treated with therapeutic preparations containing radiosensitizer Hf and RT displayed the most severe DNA damage, accompanied by increased calreticulin (CRT) exposure and high mobility group box 1 (HMGB1) release (**Figure** **5**A), which effectively improved tumor immunogenicity. Flow cytometry analysis indicated that Ft‐E64/Hf@Lipo (+) treatment couldn't affect TAM phenotype (Figure S14, Supporting Information). However, compared to the Ft/Hf@Lipo (+) group, there was a 19.8% increase in MHC‐I molecule expression on M2 TAMs after Ft‐E64/Hf@Lipo (+) treatment (Figure 5B), indicating that Ft‐E64/Hf@Lipo effectively enhanced the antigen presentation capacity of TAMs. Moreover, it could be observed that compared to the Saline (+) group, the population of tumor‐infiltrating IFN‐γ^+^CD8^+^ T cells significantly improved by 17.4% (Figure 5C). In addition, we also further analyzed the other immune cell populations in the TME. As shown in Figure S15, Supporting Information, Ft‐E64/Hf@Lipo combined RT treatment didn't influence the population of TAMs and MDSCs, while increasing the DC population. This may be attributed to the immunogenic death of tumor cells induced by RT, which releases a large amount of tumor antigens and thus recruits DCs to tumor tissue. Moreover, the Ft‐E64/Hf@Lipo (+)‐induced enhanced antigen presentation of DCs could also synergistically activate the anti‐tumor immune response (Figure S16, Supporting Information). Subsequently, the systemic immune responses were further assessed. As shown in Figure S17A,B, Supporting Information, compared with the Saline (+) group, the population of activated IFN‐γ^+^CD8^+^ T cells and Ki67^+^CD8^+^ T cells in the distant tumor tissue after RT in combination with Ft‐E64/Hf@Lipo treatment increased by 1.9‐fold and 2.1‐fold, respectively. Notably, we also observed that the proportion of effector memory T cells (Tem) in the spleen collected from CT26 tumor‐bearing mice treated with Ft‐E64/Hf@Lipo (+) significantly improved by 2.6‐fold (Figure 5D). And the serum of mice in the Ft‐E64/Hf@Lipo (+) group exhibited significantly upregulated pro‐inflammatory factors levels (IFN‐γ, IL‐6, and TNF‐α), and decreased anti‐inflammatory factor levels of IL‐10 (Figure S17C, Supporting Information). The above results collectively indicated that E64/Hf@Lipo induced a robust systemic immune response, thus resulting in distal tumor regression.

### 2.6 Ft‐E64/Hf@Lipo Enhanced Radio‐Immunotherapy Sensitizes Tumor to aPD‐L1

**Figure 6:**
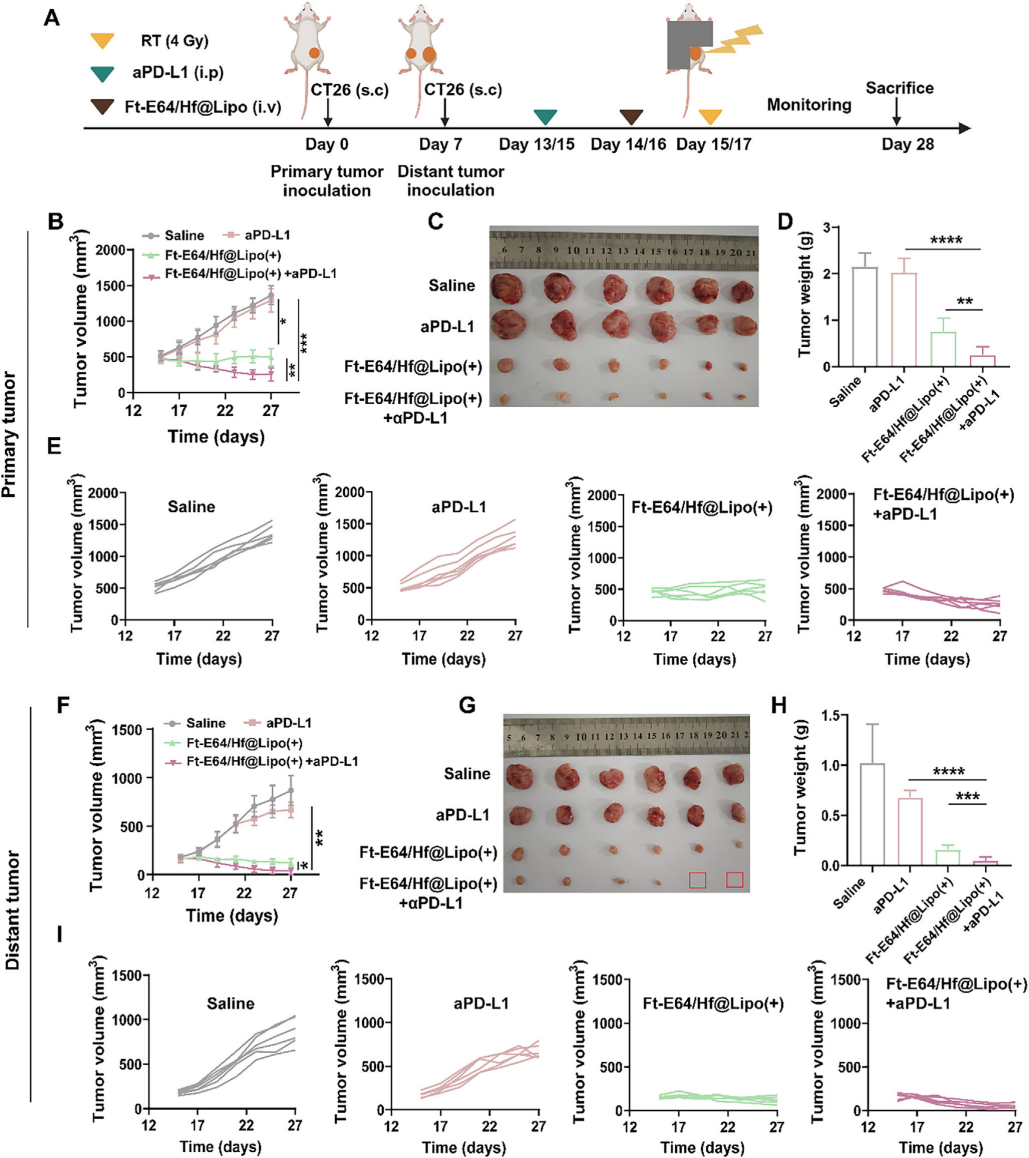
Synergistic antitumor effect of Ft‐E64/Hf@Lipo‐enhanced radioimmunotherapy and aPD‐L1 treatment in a large established CT26 tumor model. A) Schematic diagram illustrating the construction and therapeutic procedure of a CT26 large‐volume bilateral tumor‐bearing mouse model. B) Primary and F) distant tumor growth curves of CT26 large tumor‐bearing mice after various treatments (*n* = 6). Representative photos of the sacrificed C) primary and G) distant tumor on the 28th day (*n* = 6). The sacrificed D) primary and H) distant tumor weights (*n* = 6). Individual E) primary and I) distant tumor growth curves of mice after different treatments (*n* = 6). Data are means ± SD. ***p < 0.001, ****p < 0.0001 determined by Student's t‐test.

In recent years, studies have reported that RT can lead to upregulation of PD‐L1 expression in tumor cells, which greatly limits the effectiveness of radio‐immunotherapy, and accumulating evidence suggests that RT synergizes with anti‐PD‐L1 to effectively augment the antitumor responses.^[^ ^15^ ^]^ Here, through flow cytometry analysis, we also observed that the expression of PD‐L1 on tumor cells was upregulated 1.9 times after RT (Figure S18, Supporting Information). Thus, we wondered whether aPD‐L1 treatment could sensitize Ft‐E64/Hf@Lipo‐enhanced RT. Large established tumors often tend to develop complex immunosuppressive microenvironments that are resistant to immunotherapy.^[^ ^16^ ^]^ Therefore, we assessed their synergistic effect in a more advanced and larger CT26 bilateral tumor‐bearing mouse model (tumor volume, 300 mm^3^) with a low radiation dose of 4 Gy (**Figure** **6**A). As shown in Figure 6B‐E, the Ft‐E64/Hf@Lipo (+) + aPD‐L1 group effectively inhibited the growth of primary tumors with the lightest tumor mass, compared to the Ft‐E64/Hf@Lipo (+) treatment group, the primary tumor inhibition rate increased by 13.1%. Moreover, Ft‐E64/Hf@Lipo enhanced RT combined with aPD‐L1 effectively promoted the distant tumors regression with a tumor inhibition rate of 95.6% (Figure 6F–I), and the body weight of mice showed no significant change during the treatment period (Figure S19, Supporting Information), further revealing that Ft‐E64/Hf@Lipo enhanced RT in combination with aPD‐L1 effectively improved the therapeutic efficacy against large established CT26 tumor.

## 3 Conclusion

The in situ vaccine effect of radiotherapy is limited by the inefficient antigen presentation. Here, through a proteomic analysis, we found that M2 TAMs highly expressed lysosomal cysteine protease, which resulted in tumor antigen degradation. Based on the observed mechanism of TAM inhibiting antigen presentation, by skillfully utilizing the natural efferocytosis process of macrophages, we constructed an integrated nanomodulator to enable synchronous delivery of abundant tumor antigens and the small‐molecule cysteine protease inhibitor E64 to TAMs, reinvigorating TAMs to present tumor antigen and activate CD8^+^ T cells. Ft‐E64/Hf@Lipo rendered tumor cells with high immunogenicity by enhancing DNA damage and producing abundant tumor antigen, and then E64, along with the tumor antigens, was engulfed by TAMs through natural efferocytosis, endowing TAMs with a new, therapeutically beneficial property as antigen‐presenting cells. By using CT26 colorectal cancer bilateral tumor‐bearing mice as an experimental animal model, it was found that compared to RT treatment alone, the expression of MHC‐I molecule on M2 TAMs was upregulated by 23.6% and the population of activated IFN‐γ^+^CD8^+^ T cells improved by 17.4% when Ft‐E64/Hf@Lipo was combined with RT, significantly improved the antigen presentation process and T cell responses. Notably, it could synergize with PD‐L1 blockade therapy and exhibited excellent anti‐tumor effect in a large established tumor model. This study provides a powerful approach for enhancing the antigen presentation in the immunosuppressive TME and improving the treatment benefits of cancer radio‐immunotherapy.

## 4 Experimental Section

### Materials

Ferritin was purchased from Sigma‐Aldrich. Proteinase inhibitor E64, DOPC (1, 2‐Dioleoyl‐sn‐glycero‐3‐phosphocholine), DOPE (Dioleoyl phosphatidylethanolamine), SM (N‐acetyl‐D‐erythro‐sphingosylphosphorylcholine), and CH (3β‐Hydroxy‐5‐cholestene, 5‐Cholesten‐3β‐ol) were purchased from Shanghai Yuanye. Tannic acid (TA) was purchased from Aladdin. HfCl_4_ was purchased from Solarbao. DSPE‐PEG2000‐cRGD was purchased from Xi'an Ruixi. M‐CSF protein, LPS, Murine IFN‐γ, Murine IL‐2, Murine IL‐4, and Murine IL‐13 were purchased from PeproTech. ProSense 680 was purchased from PerkinElmer. DQ‐OVA was purchased from Invitrogen. FITC‐H‐2 kb, PE/Cyanine5 anti‐mouse CD11b, APC anti‐mouse CD206, APC/Cyanine7 anti‐mouse CD8a, APC anti‐mouse IFN‐γ, and PE anti‐mouse Ki67 antibodies were obtained from BioLegend. Cytofix Fix Buffer and Perm/Wash Buffer were purchased from BD Biosciences. EasySep Mouse CD8^+^ T cell Isolation Kit was obtained from Stemcell. Anti‐mouse PD‐L1 was obtained from BioXcell.

### Preparation of E64@liposome

First, HSPC, CH, and DSPE‐PEG2000 were weighed in a mass ratio of 56:39:5, respectively. The liposomes were prepared using the film dispersion method. Subsequently, the E64 and liposome were mixed in deionized water at a mass ratio of 1:1, and then subjected to ten rounds of extrusion using a microliposome extruder to obtain E64@liposome.

### DQ‐OVA Degradation Assay

The ability of M2 TAMs to degrade antigens was evaluated using the DQ‐OVA degradation assay. The M2 TAMs cultured for 12 h were treated with E64@liposome for 24 h. Subsequently, the cells were incubated with DQ‐OVA according to the operating instructions. DQ‐OVA fluorescence was detected by flow cytometry to assess the lysosomal degradative capacity of M2 TAMs. For DQ‐OVA degradation assays in vivo, after a 7 h intratumoral injection of E64@liposome, the single cell suspension of tumor tissue was treated with DQ‐OVA, and the fluorescence was quantified in M2 TAMs.

### In Vivo Antitumor Effect of E64@liposome

100 µL of CT26 cells (2×10^6^) suspended in PBS were subcutaneously injected into the mice. When the tumor volume was 100 mm^3^, mice were randomly divided into Saline and E64@liposome groups (*n* = 6 mice in each group). E64@liposome was intratumorally administered at an interval of 48 h (E64: 0.3 mg kg^−1^). The tumor volume of the mice was monitored throughout the treatment period.

### Flow Cytometry Analysis of Immune Cells

The tumors collected after E64@liposome treatments were dissected into small fragments, and 0.2 g of random tumor tissue samples was obtained. A tumor dissociation kit was utilized to prepare a single tumor cell suspension. For detection of the MHC‐I expression on M2 TAMs, the cells were stained with APC/Cyanine 7‐CD45, PE/Cyanine 5‐F4/80, PE‐CD206, and FITC‐H‐2 kb antibodies. To investigate the activation of CD8^+^ T cells. The cells were subjected to surface staining using PerCP/Cyanine 5.5‐CD45, FITC‐CD3, and APC/Cyanine 7‐CD8a antibodies. Then the fixed cells were further stained with PerCP/Cyanine 7‐IFN‐γ, followed by flow cytometry analysis to detect the number of activated CD8^+^ T cells.

### Preparation of Ft‐E64

2 mg of Ft and 2 mg of E64 were weighed and dissolved in 1 mL of deionized water, respectively. The solutions were mixed thoroughly by ultrasound, and then magnetically stirred in a water bath at 60 °C for 2.5 h. Free E64 was removed by centrifugation at 3000 rpm after the reaction, and Ft‐E64 nanoparticles were obtained.

### Preparation of Ft‐E64/Hf

2 mg of Ft‐E64 dissolved in 1 mL of deionized water, 1 mg of TA dissolved in 50 µL of deionized water and 0.6 mg of HfCl_4_ dissolved in 200 µL of deionized water was put into a 50 mL of round‐bottom flask and reacted in a water bath at 37 °C under magnetic stirring for 6 h. the Ft‐E64/Hf nanoparticles with an average particle size of ≈90 nm were obtained by centrifugation. Notably, the size of the self‐assembled Ft‐E64/Hf nanoparticles can be controlled by regulating the feeding ratio (Ft‐E64/TA/Hf) and the reaction time.

### Preparation of Ft‐E64/Hf@Lipo

First, the RGD‐functionalized fusogenic liposomes were prepared using the film dispersion method. DOPC, DOPE, SM, and CH were weighed in a mass ratio of 7:6:3:4, respectively. DSPE‐PEG2000‐cRGD peptide (20 mg) was dissolved in 1 mL of N, N‐dimethylformamide, and then 100 µL of this solution was added to the fusogenic liposome‐forming solution obtained from the previous steps. After reaction at 4 °C for 12 h, cRGD peptide‐functionalized liposomes (Lipo) were obtained. Subsequently, the Ft‐E64/Hf and Lipo were mixed in deionized water at a mass ratio of 1:1, and then subjected to ten rounds of extrusion using a microliposome extruder to obtain Ft‐E64/Hf@Lipo.

### In Vitro Detection of Hf Release

The Ft‐E64/Hf was dissolved in the GSH solution at a concentration of 0, 1, and 10 mM, respectively. Following an incubation period of 48 h, the supernatant was collected to detect the Hf release using ICP‐MS.

### Cumulative Drug Release at Different pH Conditions

To investigate the E64 release behavior under an acidic environment, Ft‐F/Hf was prepared by replacing E64 with fluorescein FITC. Equal amounts of Ft‐F/Hf were placed in PBS solutions with different pH values at 37 °C. The absorbance of supernatant after different incubation times was measured, and the released drug content was determined.

### Cell Culture

CT26 cells and RAW264.7 obtained from American Type Culture Collection (ATCC) were cultured in RPMI‐1640 medium containing 10% fetal bovine serum (FBS) and DMEM medium containing 10% FBS at 37 °C under 5% CO_2_, respectively.

### Membrane Fusion Assay

To investigate the fusion process of Lipo and CT26 cells, DiO‐labeled Lipo was prepared by incubating 10 µM DiO with Lipo for 30 min. Subsequently, the DiO‐labeled Lipo was incubated with DiI‐labeled CT26 cells in the dark for different time intervals. After discarding the medium, the cells were treated with a fixative solution containing 4% paraformaldehyde and then stained with DAPI for 5 min. The fusion process between CT26 cells and Lipo was visualized using confocal laser scanning microscopy.

### Detection of DNA Damage

CT26 cells were divided into six groups as follows: 1) PBS group; 2) Ft‐E64/Hf@Lipo group; 3) RT group, denoted as PBS (+); 4) Ft‐E64@Lipo + RT group, denoted as Ft‐E64@Lipo (+); 5) Ft/Hf@Lipo + RT group, denoted as Ft/Hf@Lipo (+); and 6) Ft‐E64/Hf@Lipo + RT group, denoted as Ft‐E64/Hf@Lipo (+). The cells were incubated with Ft‐E64/Hf@Lipo, Ft‐E64@Lipo, or Ft/Hf@Lipo‐containing medium for 6 h. Subsequently, CT26 cells in (3), (4), (5), and (6) groups were exposed to 6 Gy irradiation and cultured for another 48 h. The cells fixed with paraformaldehyde fixative were incubated with γ‐H2AX antibody for 30 min, followed by DAPI staining for 5 min. Finally, CLSM was employed to acquire images.

### Detection of Cell Apoptosis

The CT26 cells were cultured and then treated with the drug‐containing culture medium. After 6 h of co‐incubation, the cells were treated with 6 Gy irradiation. Then the cells were collected after 48 h of culture and treated with the Apoptosis assay Kit according to the operating instructions for flow cytometry analysis.

### Macrophages Engulf Apoptotic Tumor Cells

CT26 cells were incubated with Ft‐E64/Hf@Lipo for 6 h, followed by 6 Gy irradiation. After culturing for 48 h, the cells were collected and stained with 10 µL Annexin V‐FITC for 30 min. Subsequently, the obtained Annexin V‐FITC‐labeled apoptotic cells were co‐incubated with M2 TAMs for 1 h and then washed with PBS twice after discarding the supernatant. The obtained M2 TAMs were labeled with PE anti‐mouse F4/80 antibody and PE/Cyanine5 anti‐mouse CD206 antibody and then analyzed by flow cytometry.

### Detection of Cysteine Protease Activity

The CT26 cells were incubated with Ft/Hf@Lipo or Ft‐64/Hf@Lipo for 6 h, and then exposed to an X‐ray irradiator at a dose of 6 Gy. After culturing for 48 h, the CT26 cells were incubated with pre‐polarized M2 TAMs for 4 h. The supernatant was removed. Subsequently, the harvested M2 cells were treated with ProSense 680 (1 µM) for 6 h at 37 °C, and then the fluorescence signals of M2 TAMs were quantified using flow cytometry.

### The Expression of MHC‐I Molecule on M2 TAMs

The M2 TAMs were treated with apoptotic CT26 cells after various treatments for 24 h. The supernatant was discarded, and the cells were incubated with FITC anti‐mouse H‐2Kb antibody. The MHC‐I molecules on M2 TAMs were quantified using flow cytometry.

### CD8+ T Cells Activation

M2 TAMs were co‐incubated with apoptotic CT26 cells pretreated with various treatments for 24 h. The supernatant was discarded, and then M2 TAMs were collected. To assess the activation of CD8^+^ T cells, pretreated M2 TAMs were co‐cultured with CD8^+^ T cells isolated from the spleen at a ratio of 1:1 for a period of 3 days. Subsequently, the cells were harvested and incubated with BD GolgiPlug for 2 h at 37 °C. Then the cells were stained with anti‐mouse CD8a antibody for 15 min to obtain activation labeling. After being treated with BD Cytofix fixation buffer for 20 min, the permeable cells were stained with APC anti‐mouse IFN‐γ antibody while being treated with BD Perm/Wash Buffer. Finally, IFN‐γ^+^CD8^+^ T cells were quantified by flow cytometry.

### Mice

Female BALB/c mice (18–20 g) were purchased from Zhejiang Vital River Laboratory Animal Technology Co., Ltd. The license number is SCXK (zhe) 2019‐0001.

### The Ethical Approval Statement

All the animal experiments were performed in accordance with the guidelines of the Regional Ethics Committee for Animal Experiments and Zhengzhou University Institutional Animal Care and Use Committee (24‐IACUC‐Y129).

### Distribution of Ft‐E64 across Different Cell Populations

CT26 tumor‐bearing mice were intravenously administered Saline and Ft‐E64/Hf@Lipo. At 12 h postinjection, the tumor in the Ft‐E64/Hf@Lipo treatment group was treated with 6 Gy irradiation. After 24 h, the tumors with different treatments were collected, and the single‐cell suspensions obtained with a Tumor Dissociation Kit were then stained with the corresponding antibodies. TAMs, DCs, T cells, CAF cells, and MDSCs were sorted by flow cytometry, respectively. The sorted cells were counted, and the Fe content in different cells (1×10^6^) was measured by ICP‐MS.

### In Vivo Antitumor Activity of Ft‐E64/Hf@Lipo Combined RT

100 µL of CT26 cells (2×10^6^) were subcutaneously injected into the right hind limb of mice to construct the primary tumors. Four days after the inoculation of primary tumors, 100 µL of CT26 cells (2×10^6^) suspended in PBS was administered into the left hind limb as distal tumors. When the primary tumor volume was 100 mm^3^, mice were randomly divided into the following groups (*n* = 6 mice in each group): 1) the mice treated with saline, Saline group; 2) the mice treated with Ft‐E64/Hf@Lipo, Ft‐E64/Hf@Lipo group; 3) the mice treated with RT, Saline (+) group; 4) the mice treated with Ft/Hf@Lipo plus RT, Ft/Hf@Lipo (+) group; and 5) the mice treated with Ft‐E64/Hf@Lipo plus RT, Ft‐E64/Hf@Lipo (+) group. Various preparations were administered via the tail vein at an equal dose of 6.2 mg kg^−1^ for Hf and 0.3 mg kg^−1^ for E64. At 12 h postinjection, the primary tumor tissues of mice in groups (3), (4), and (5) were treated with 6 Gy irradiation, while the distant tumors were not irradiated. The treatment process was performed for two cycles. The tumor volume and body weight of the mice were monitored, and both the primary and distal tumor tissues were collected post‐treatment for in vivo anti‐tumor mechanism analysis.

### Systemic Immune Assessment

The spleen of tumor‐bearing mice after various treatments was collected, ground, and then centrifuged. The production was incubated in RBC lysis buffer and filtered to obtain a single‐cell suspension. The cells were stained with FITC‐CD3, APC/Cyanine 7‐CD8a, APC‐CD44, and PE/Cyanine 7‐CD62L antibodies. Then, flow cytometry analysis was performed to determine CD3^+^CD8a^+^CD44^+^CD62L^−^ cells.

### Synergistic Antitumor Effect of Ft‐E64/Hf@Lipo‐enhanced RT and aPD‐L1 Treatment in a Large CT26 Tumor Model

To evaluate the synergistic effect of Ft‐E64/Hf@Lipo combined RT and anti‐PD‐L1 therapy, a bilateral tumor model was established. When the primary tumor reached 300 mm^3^, mice were randomly divided into the following four groups (*n* = 6): 1) Saline; 2) aPD‐L1; 3) Ft‐E64/Hf@Lipo (+); 4) Ft‐E64/Hf@Lipo (+) + aPD‐L1, “+” represents RT. The mice in groups 2 and 4 were intraperitoneally injected with aPD‐L1 (5 mg kg^−1^). The mice in groups three and four were injected with Ft‐E64/Hf@Lipo via the tail vein (Hf concentration: 6.2 mg kg^−1^). After 24 h, the primary tumor was treated with local RT at a dose of 4 Gy, while the left distant tumor remained untreated. The treatment process was performed for two cycles, and the tumor volumes were monitored to evaluate the antitumor effect.

### Statistical Analysis

All data presented the means ± standard deviation (SD). *n* = 5 for proteomic analysis; *n* = 6 for in vitro CT26 cell viability measure, in vivo tumor volume and tumor weight detection, as well as mice body weight detection; *n* = 3 for the other quantitative experiments. Statistical analysis was performed via a one‐tailed Student's t‐test to test the significant difference between the sample groups with GraphPad Prism version 8.0 Software. Statistical significance was indicated as *p < 0.05, **p < 0.01, ***p < 0.001 and ****p < 0.0001.

## Conflict of Interest

The authors declare no conflict of interest.

## Author Contributions

X.Z. and M.L. contributed equally to this work. X.Z. conceived and designed the experiments, wrote the manuscript, supervised the entire project, and provided the research funding in the initial manuscript submitted. M.L. performed the experiments with assistance from Jun Li and Yueying Han in the initial manuscript submitted. Y.G. performed the experiments during the manuscript revision. Z.Z. revised the manuscript in the initial manuscript submitted. J.S. revised the methods sections and provided the partial research funding in the initial manuscript submitted. C.‐Y.J. and J.L. conceived this project, revised the manuscript, and supervised the entire project in the initial manuscript submitted. In addition, J.L. also provided the research funding for the initial manuscript submitted. P.S. provided the research funding, designed the experiments, performed in vivo experiments, revised the manuscript, and supervised the entire project during the manuscript revision.

## Supporting information



Supporting Information

## Data Availability

The data that support the findings of this study are available from the corresponding author upon reasonable request.
