# Supplementary material for: A Nanomodulator Enhances Radiotherapy‐Induced In Situ Cancer Vaccine by Promoting Antigen‐Presenting of Tumor‐Associated Macrophage
Source: Adv Sci (Weinh). 2025 Aug 5;12(40):e02876. doi: 10.1002/advs.202502876 (PMC12561198; doi:10.1002/advs.202502876)
Supplement: Supplementary file 1 — Supporting Information [file ADVS-12-e02876-s001.docx]

Supporting Information

A Nanomodulator Enhances Radiotherapy-Induced In Situ Cancer Vaccine by Promoting Antigen-Presenting of Tumor-Associated Macrophage

*Xiu Zhao^‡^, Mengli Li^‡^, Jun Li, Yueying Han, Yu Gong, Zhenzhong Zhang, Jinjin Shi, Cheng-Yun Jin*, Junjie Liu*, and Pilei Si**


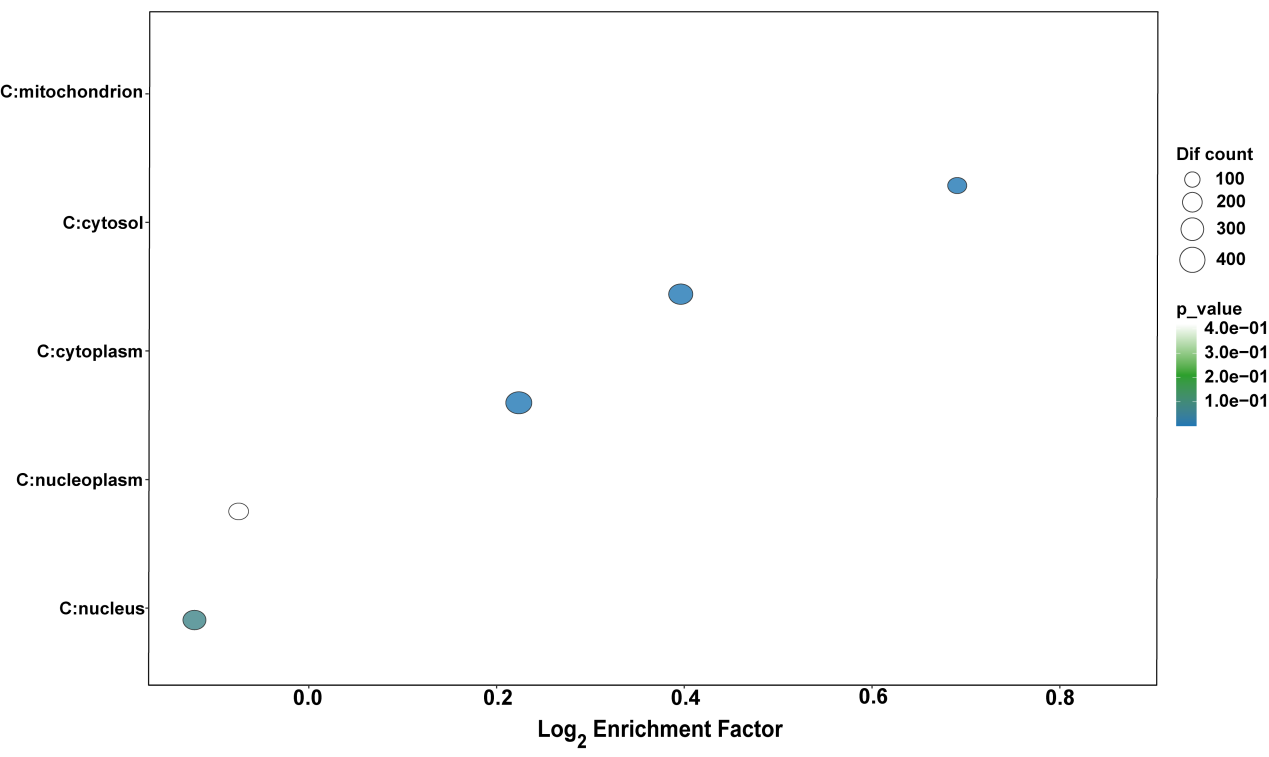


**Figure S1.**  Top five pathways from gene ontology (GO) analysis of proteins elevated in M2 BMDMs (*P*< 0.05, Fisher’s exact test with Benjamini-Hochberg correction) (*n* = 5).


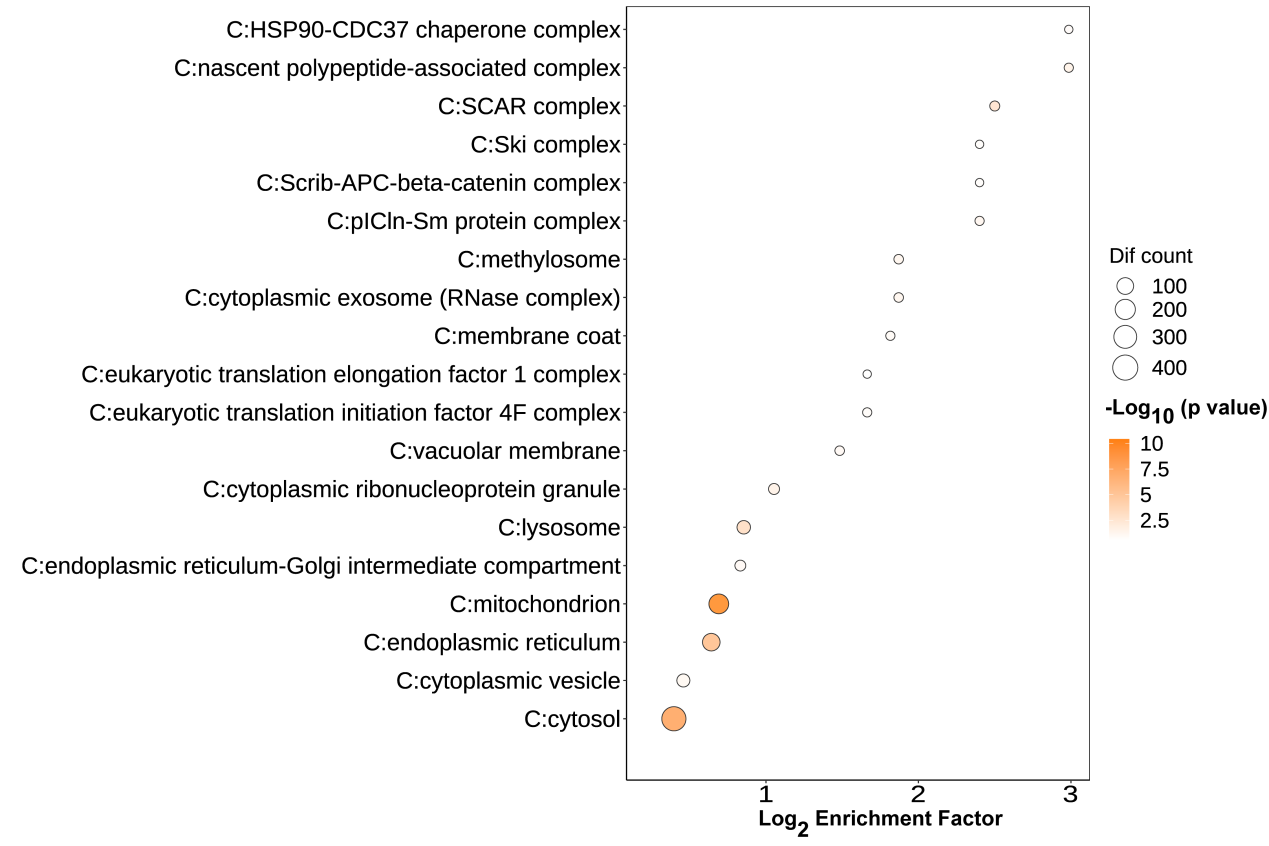


**Figure S2.** Top pathways from gene ontology (GO) analysis of proteins differentially expressed in the cytoplasm (*P*< 0.05, Fisher’s exact test with Benjamini-Hochberg correction) (*n* = 5).


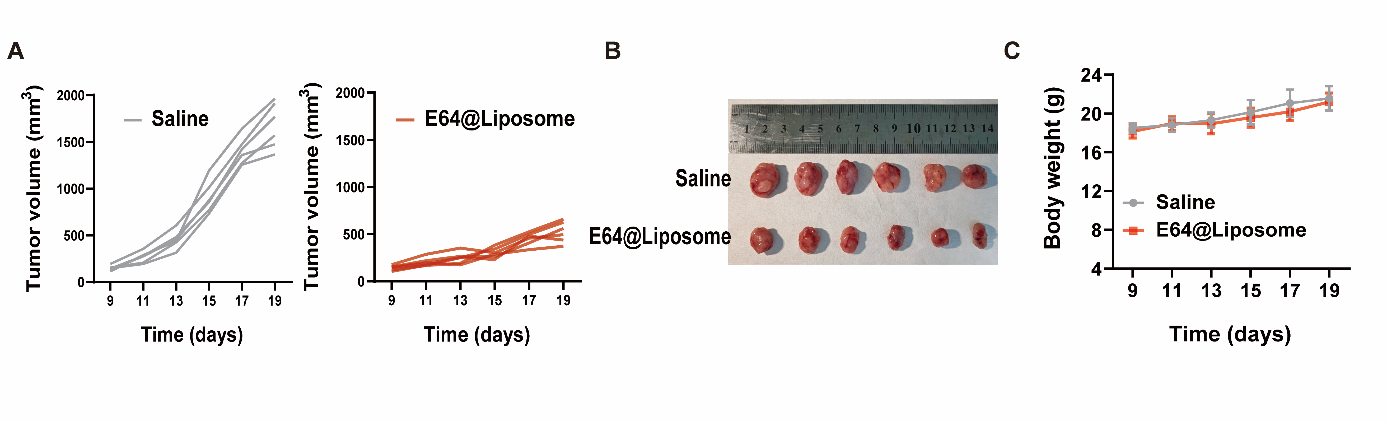


**Figure S3.** A) Individual tumor growth curves of mice after different treatments (*n* = 6). B) The sacrificed tumor weights on the 19th day (*n*= 6). C) Body weight monitoring of tumor-bearing mice with Saline and E64@Liposome treatments (*n* = 6). Data are means ± SD.


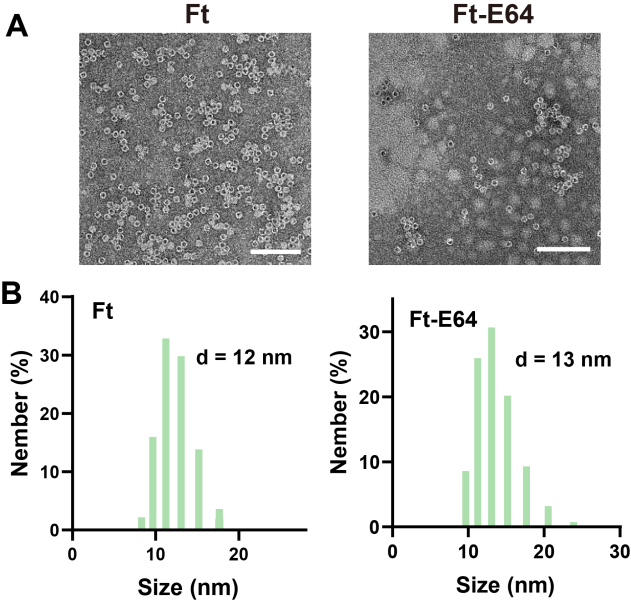


**Figure S4.** A) TEM images of Ft and Ft-E64. Scale bars: 100 nm. B) DLS analysis of Ft and Ft-E64.


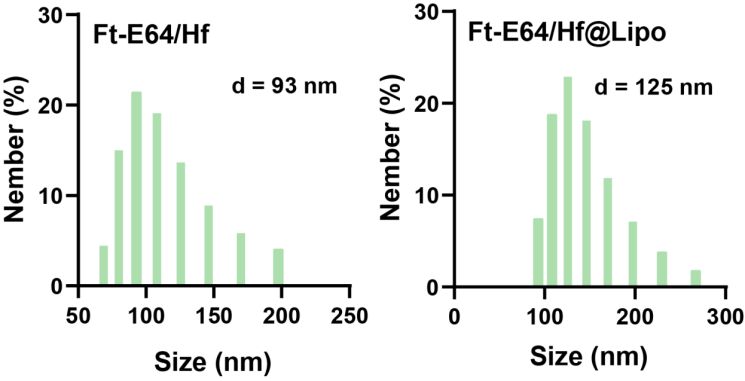


**Figure S5.** DLS analysis of Ft-E64/Hf and Ft-E64/Hf@Lipo.


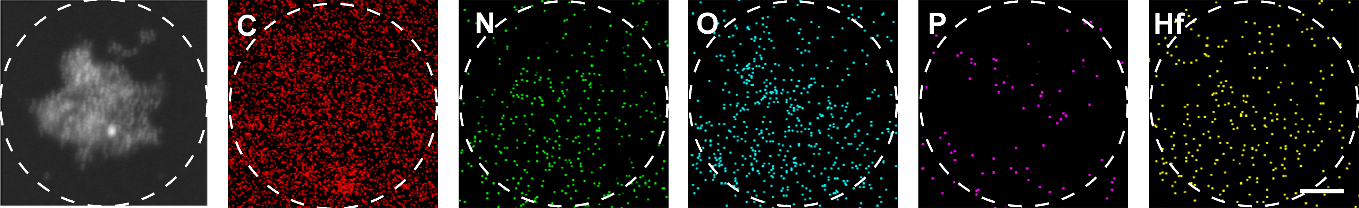

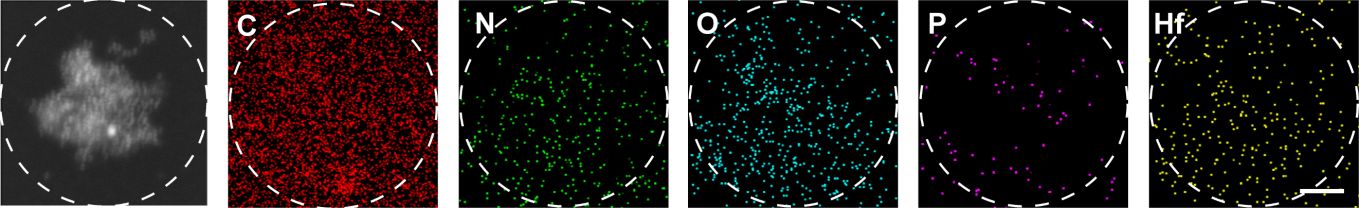


**Figure S6.** TEM mapping analysis of Ft-E64/Hf. Scale bar: 50 nm.


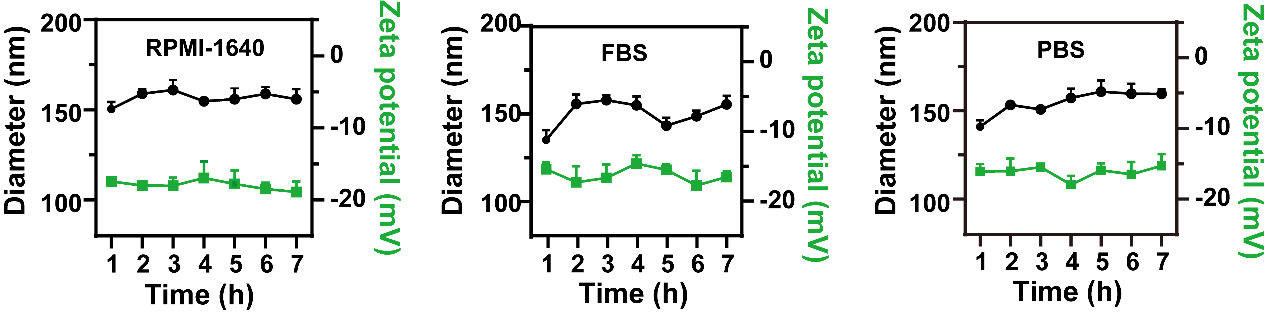


**Figure S7.** Stability analysis of Ft-E64/Hf@Lipo in PBS, RPMI-1640, and fetal bovine serum (*n* = 3). Data are means ± SD.

**
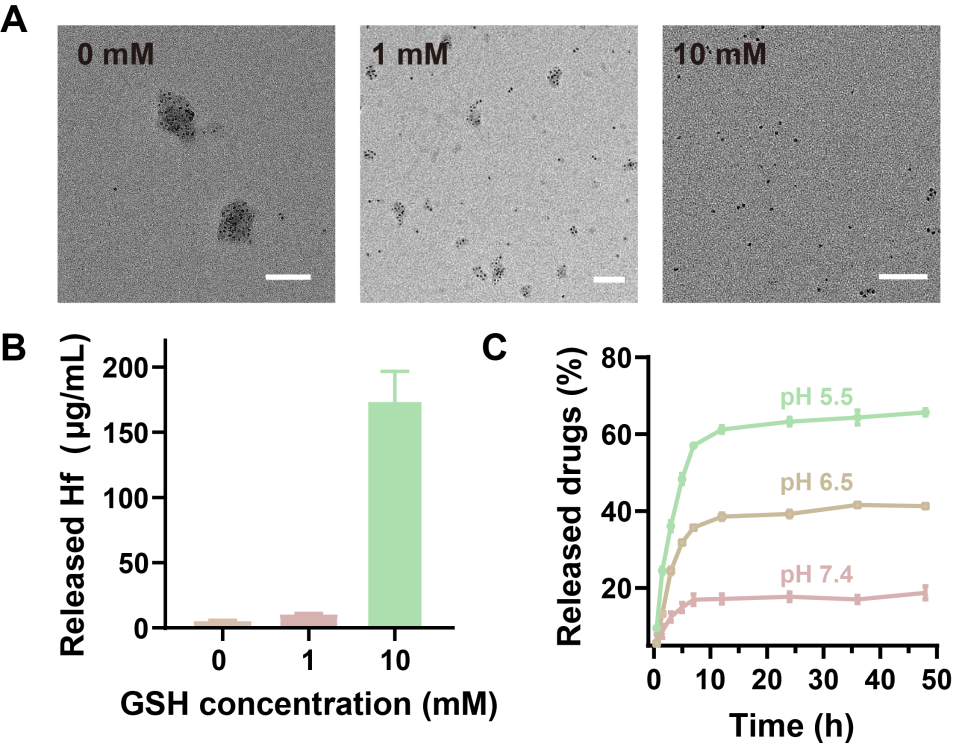
**

**Figure S8.** A) TEM images of Ft-E64/Hf after treatment with different concentrations of GSH. Scale bars: 100 nm. B) Hf release amount from Ft-E64/Hf under different concentrations of GSH as measured by ICP-MS (*n* = 3). C) Drug cumulative release profile from Ft-E64 at different pH values (*n* = 3). Data are means ± SD.


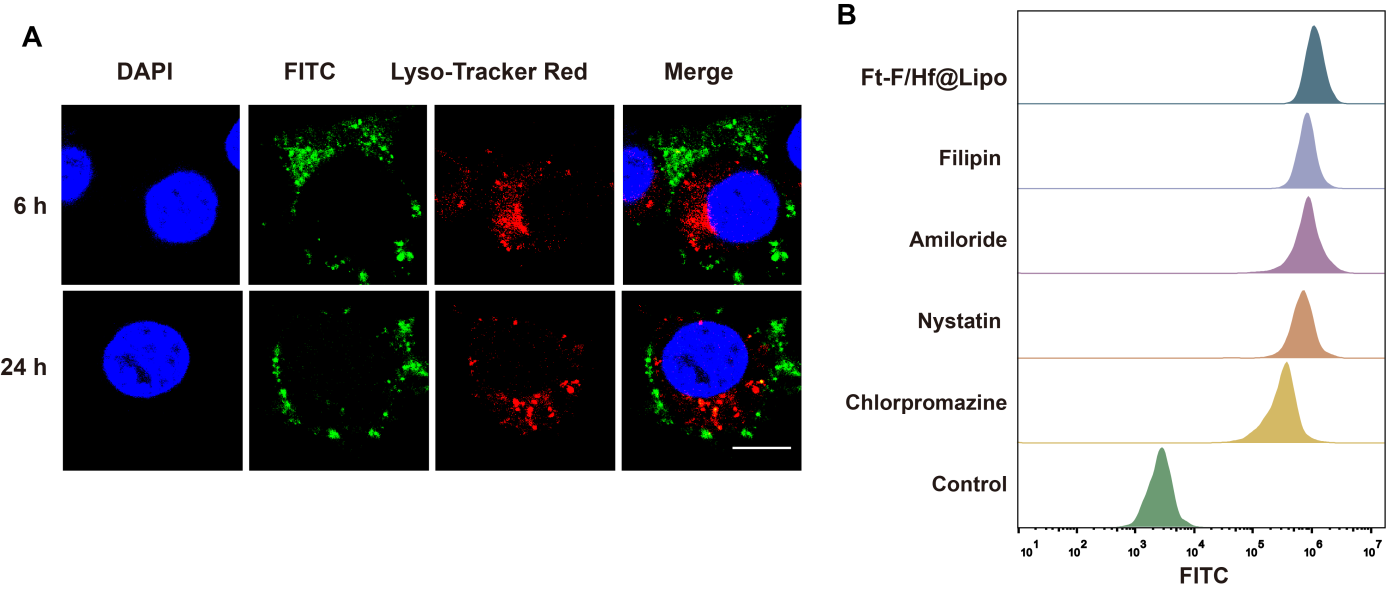


**Figure S9.**  A) Intracellular distribution of Ft-F/Hf at 6 h and 24 h after cellular uptake. Scale bar: 10 μm. B) Flow cytometry analysis of Ft-F/Hf@Lipo uptake after different pre-treatments.

**
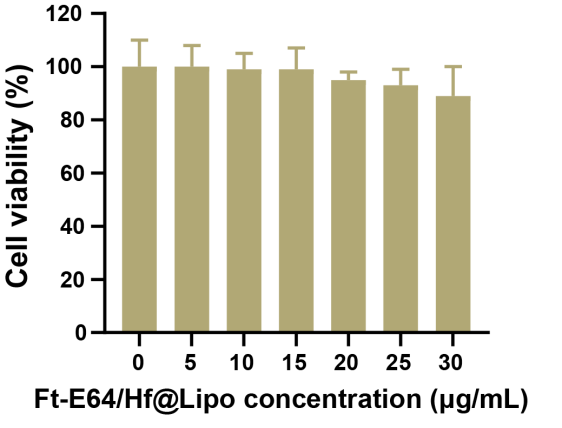
**

**Figure S10.** The survival rate of CT26 cells treated with different concentrations of Ft-E64/Hf@Lipo (*n* = 6). Data are means ± SD.


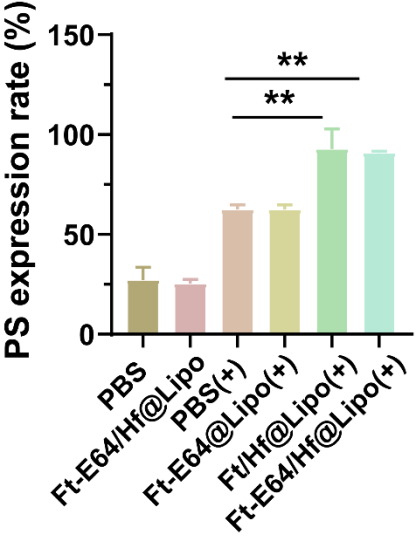


**Figure S11.** Expression level of phosphatidylserine in CT26 cells after various treatments (*n* = 3). Data are means ± SD. ***P* < 0.01 determined by Student’s t-test.


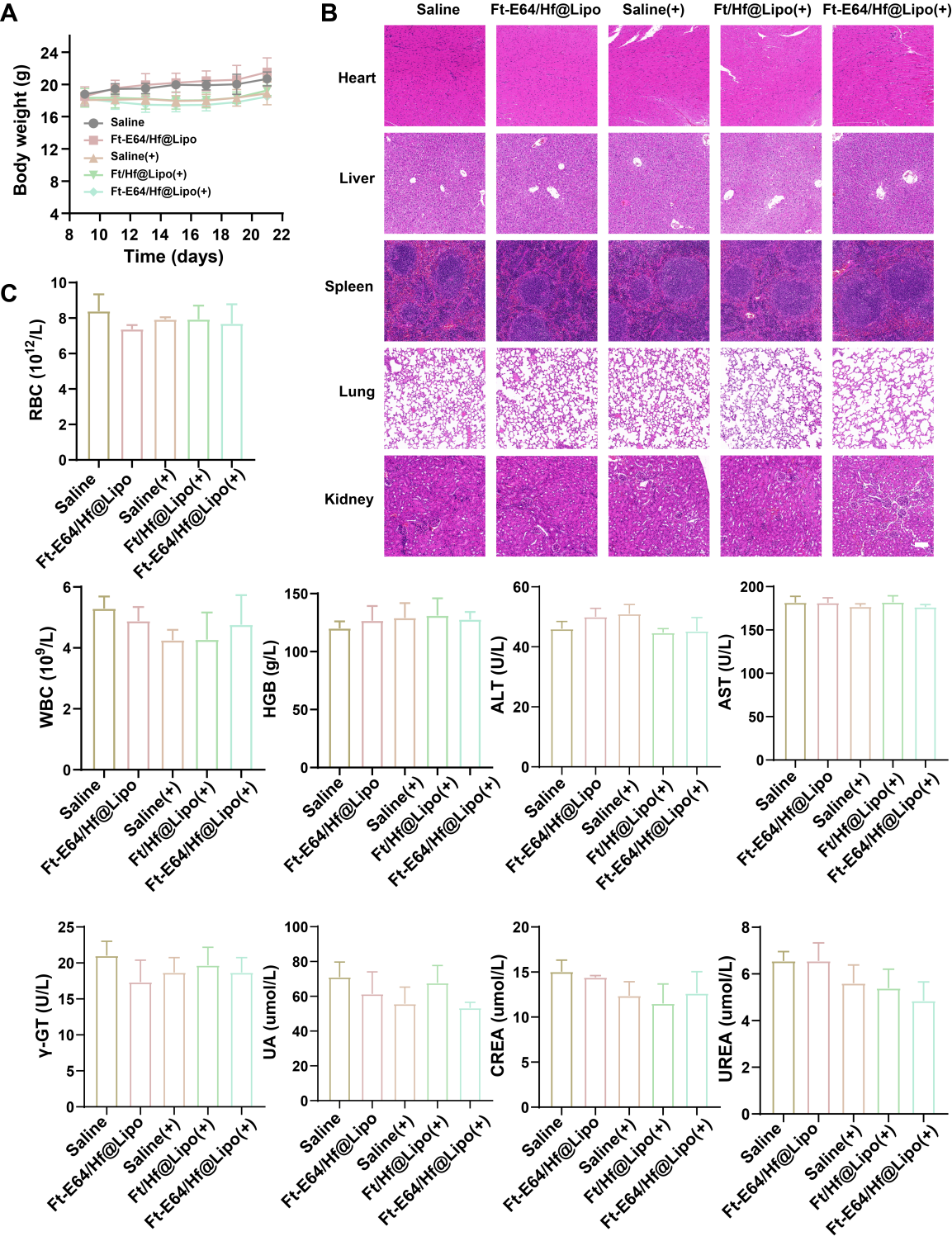


**Figure S12.** Biosafety analysis of Ft-E64/Hf@Lipo. A) Body weight of tumor-bearing mice after various treatments (*n* = 6). B) H&E staining of main organs in mice after various treatments. C) Blood routine and blood biochemical tests of tumor-bearing mice after various treatments (*n* = 3). Data are means ± SD.


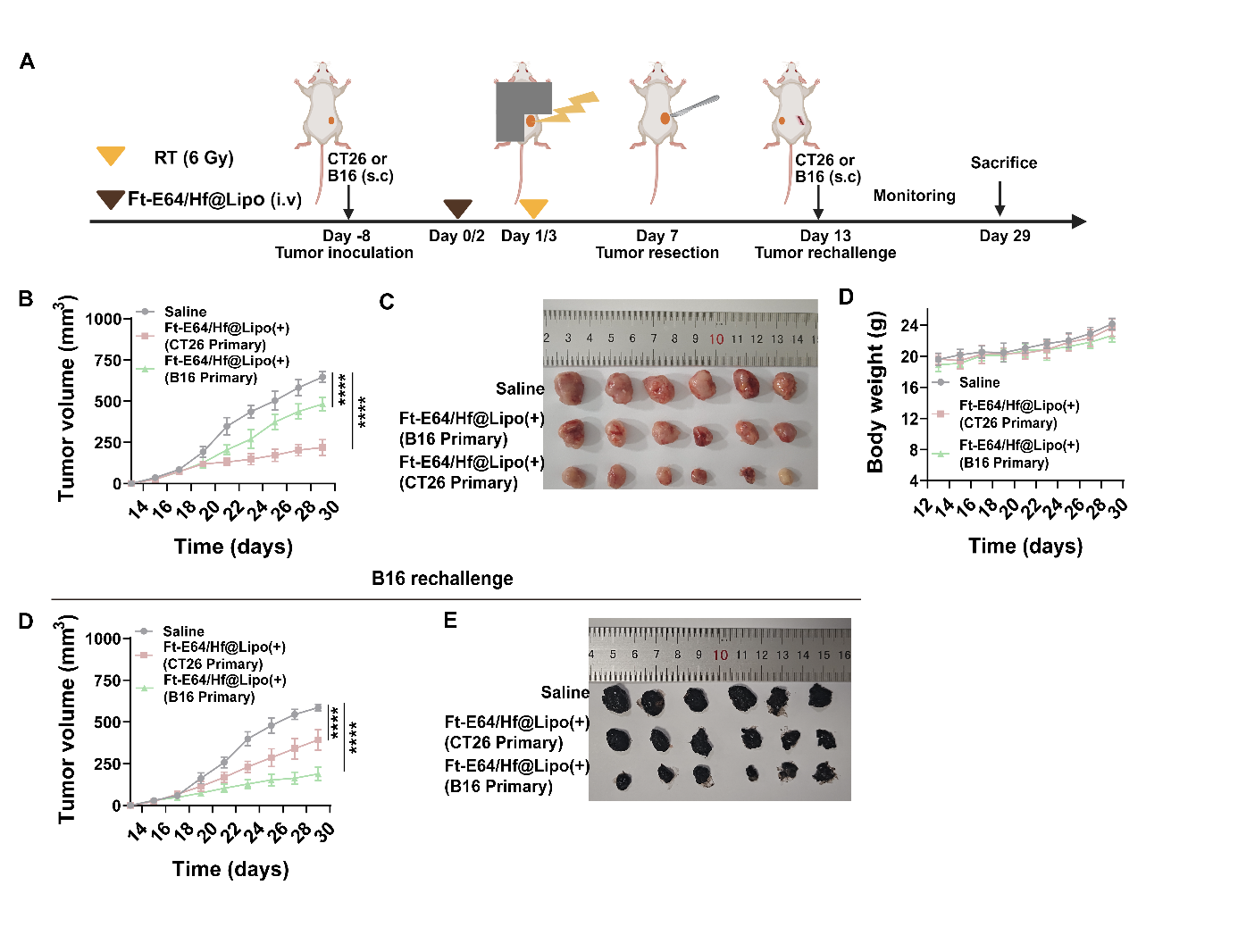


**Figure S13.** A) Schematic diagram illustrating the construction of a CT26 tumor rechallenge model. B) CT26 tumor growth curves of mice after various treatments (*n* = 6). C) The representative photos of the sacrificed CT26 tumor on the 29th day (*n* = 6). D) Body weight change of tumor-bearing mice after various treatments (*n* = 6). Data are means ± SD. *****P* < 0.0001 determined by Student’s t-test.


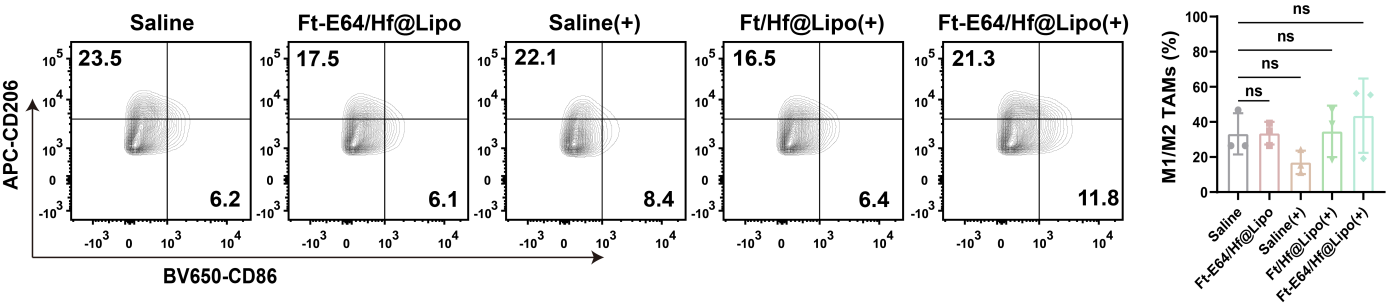


**Figure S14.** Representative flow cytometry images and the relative quantification analysis of the M1/M2 TAMs ratio in the primary tumor after different treatments (*n* = 3). Data are means ± SD.


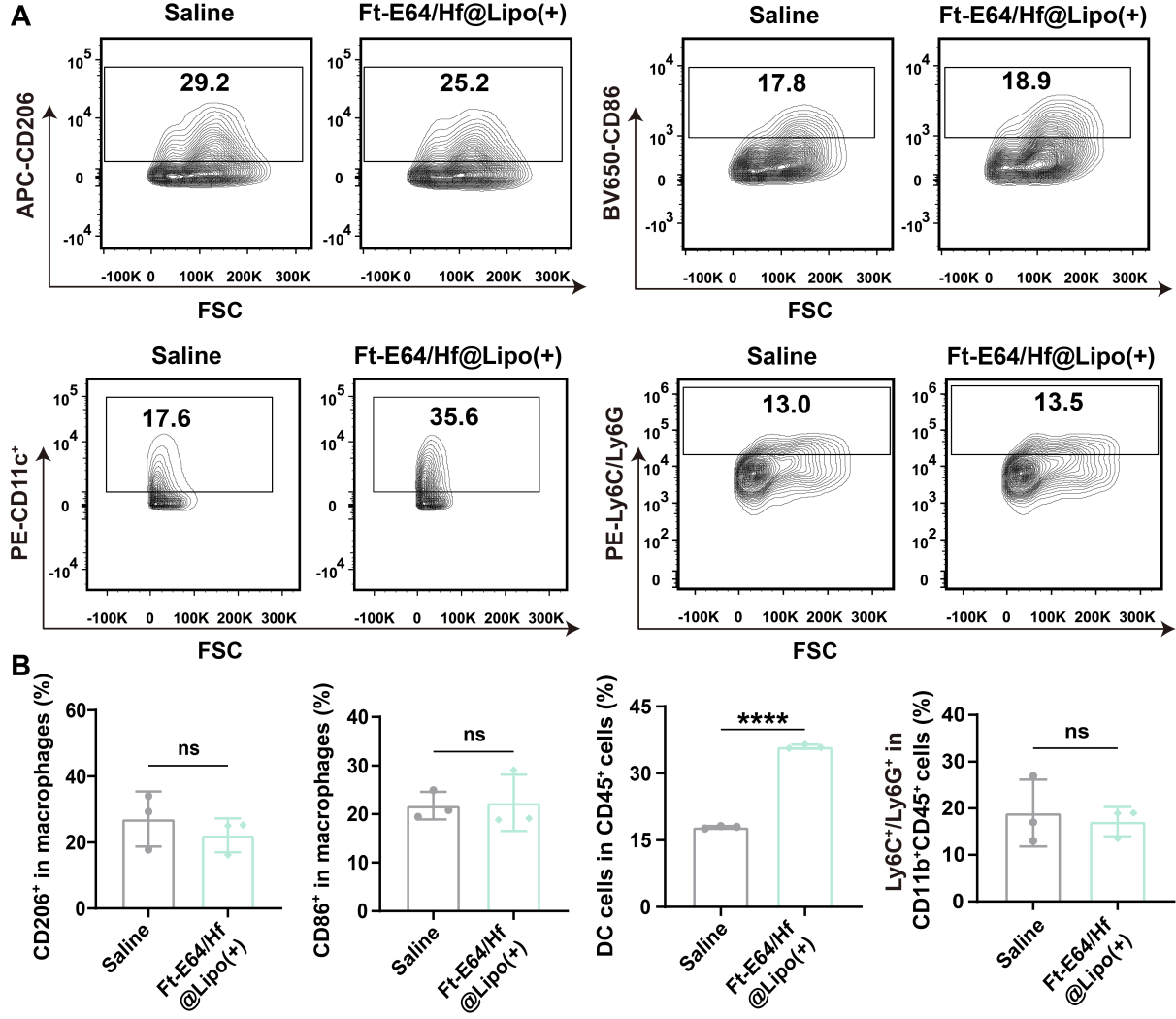


**Figure S15.** A) Representative flow cytometry images and B) the relative quantification analysis (*n* = 3) of immune cell populations in the TME after in situ vaccine treatments. Data are means ± SD. *****P* < 0.0001 determined by Student’s t-test.


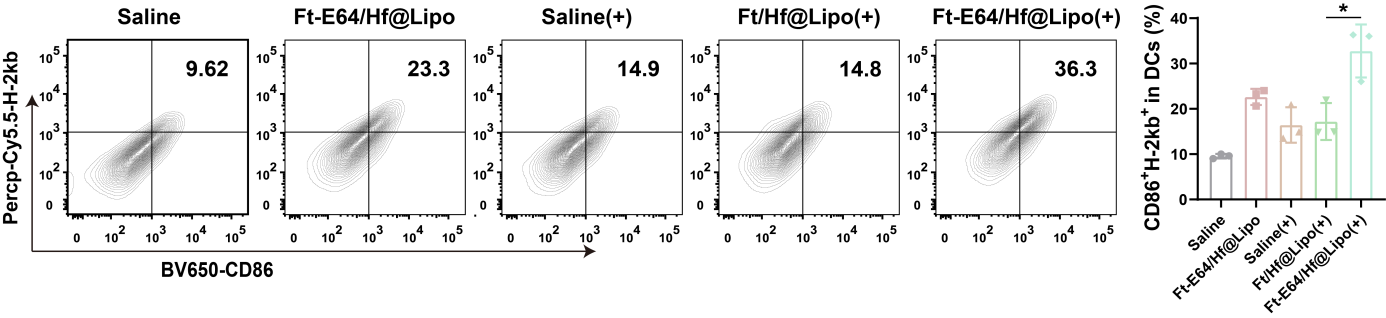


**Figure S16.** Representative flow cytometry images and the relative quantification analysis of the DCs in the tumor tissue after different treatments (*n* = 3). Data are means ± SD. **P* < 0.1 determined by Student’s t-test.


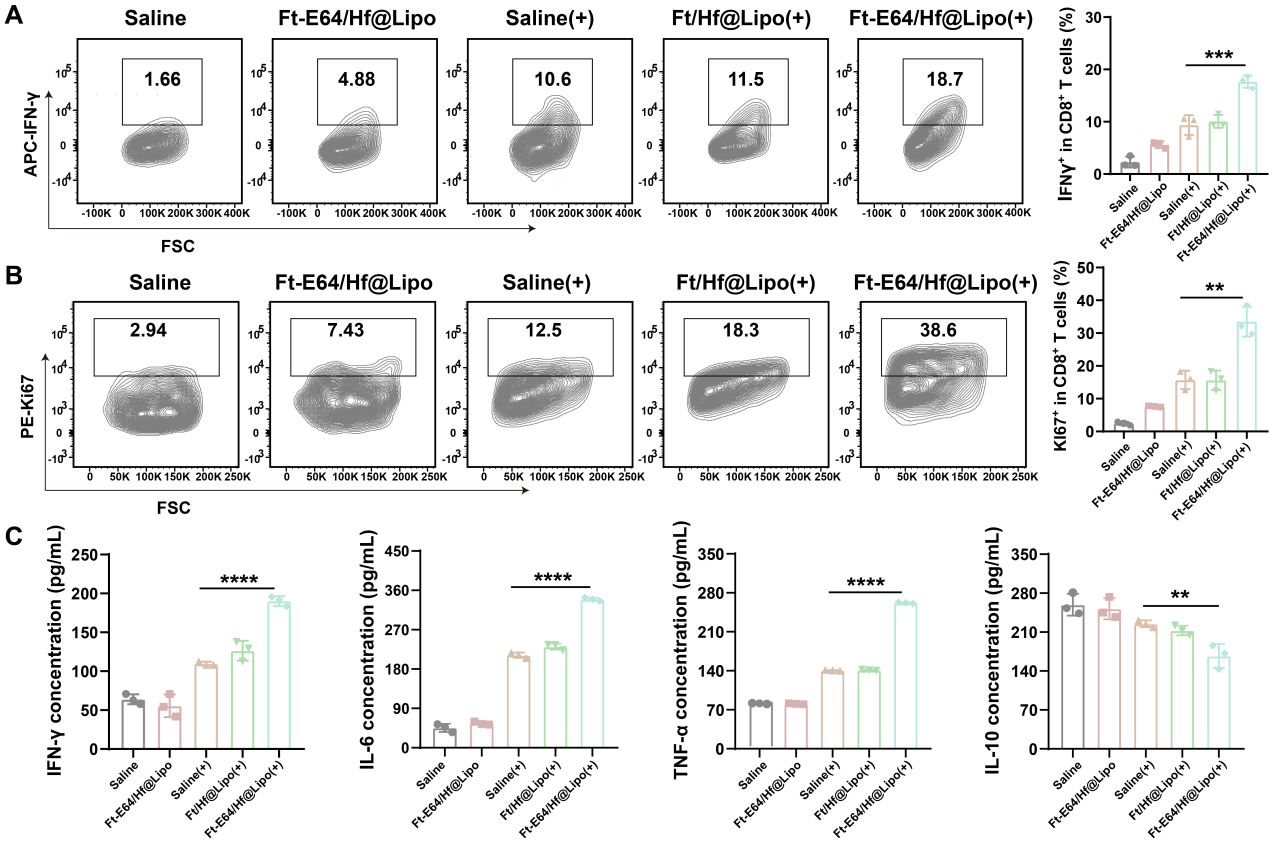


**Figure S17.** Representative flow cytometry images and the relative quantification analysis (*n* = 3) of the (A) CD8^+^ T cells activation (CD45^+^CD3^+^CD8a^+^IFN-γ^+^) and (B) CD8^+^ T cells proliferation (CD45^+^CD3^+^CD8a^+^Ki67^+^) in distant tumor. C) Cytokine levels of IFN-γ, IL-6, TNF-α, and IL-10 in the serum of mice after different treatments (*n* = 3). Data are means ± SD. ***P* < 0.01, ****P* < 0.001, *****P* < 0.0001 determined by Student’s t-test.


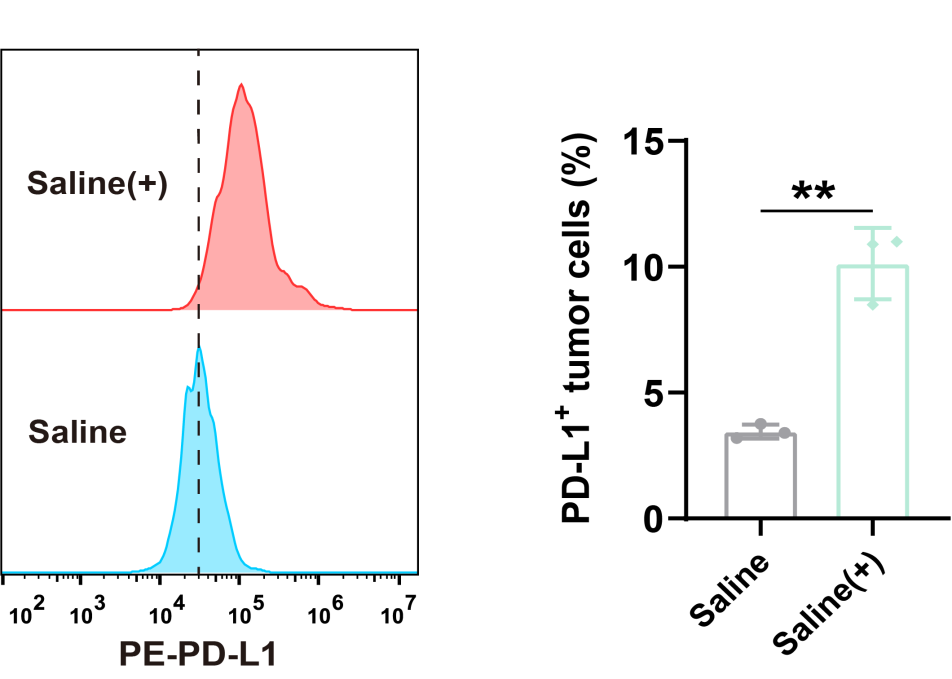


**Figure S18.** Representative flow cytometry image and the quantification analysis of PD-L1^+^ tumor cells (*n* = 3). Data are means ± SD. ***P* < 0.01 determined by Student’s t-test.


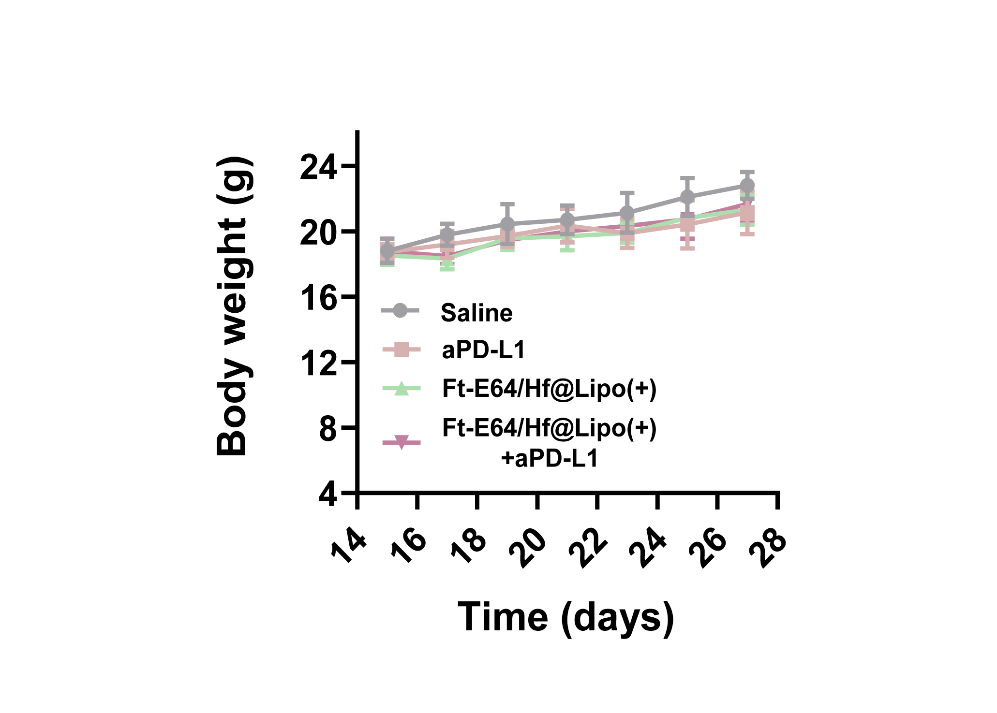


**Figure S19.** Body weight monitoring of mice after different treatments in the large established CT26 tumor model (*n* = 6). Data are means ± SD.
